# Dual‐Chamber One‐Step Molding Actuator with Straight‐Curved Crease Prismatic Design for Large‐Range Motions and Structural Stability

**DOI:** 10.1002/advs.202514441

**Published:** 2025-11-20

**Authors:** Qinlin Tan, Yanlin Chen, Meng Zhou, Shuk Fan Tong, Sicong Liu, Raymond Kai‐Yu Tong

**Affiliations:** ^1^ Department of Biomedical Engineering The Chinese University of Hong Kong Hong Kong SAR China; ^2^ Department of Mechanical and Automation Engineering The Chinese University of Hong Kong Hong Kong, SAR China; ^3^ Sino‐German College of Intelligent Manufacturing Shenzhen Technology University Pingshan 518118 China

**Keywords:** one‐step molding, origami, soft actuator, soft robot

## Abstract

Soft actuators are gaining attention for their exceptional deformability and adaptability to meet the diverse demands of practical soft robotics applications. However, soft actuators with pneumatic actuation often struggle to simultaneously achieve large‐range motions and structural stability. Addressing this challenge, a dual‐chamber straight‐curved crease origami prismatic (SCOP) actuator is proposed that introduces anisotropic stiffness through origami‐inspired straight‐curved creases and a foldable partition, enabling multidimensional motions including elongation and contraction, with an axial displacement reaching 106.7% of the actuator's original length (elongation 51.1% and contraction 55.6%), over 160° bidirectional bending, and a 209% increase in structural stability compared to curve crease bellows. The actuator is fabricated through one‐step molding using a single material, and its principles, modeling, and design are systematically analyzed. The versatility and feasibility of the SCOP actuator are validated through its integrations into a wearable assistive device, a soft gripper, and a single‐actuator crawling robot, which leverage various combinations of the SCOP's actuation modes to meet diverse motion requirements. This design provides a high‐performance and practical solution for soft robotic systems requiring large‐range, multidirectional motions with enhanced strength and reliability.

## Graphical abstract

A dual‐chamber origami prismatic actuator is designed by integrating straight‐curved creases with folded partitions, enabling large‐range, multidirectional motions and enhanced structural stability. The actuator exhibits anisotropic stiffness and multiple actuation modes, and is fabricated via single‐material, one‐step molding. Its design supports diverse robotic applications, including assistive devices, a soft gripper, and a crawling robot.

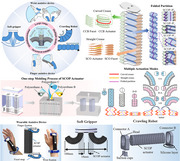

## 1 Introduction

In recent years, soft robotics technology has garnered increasing attention. The exceptional environmental adaptability and deformability grant soft robots promising potential for applications in human‐robot interaction, dexterous manipulation, and safe operations in unstructured environments.^[^ ^1^, ^2^, ^3^ ^]^ As a crucial component of soft robots, soft pneumatic actuators (SPA) are characterized by inherent compliance, complex motions, lightweight and low cost.^[^ ^4^, ^5^ ^]^ Developments in soft robotics are propelled by the progress of soft actuators, with researchers continuously striving to create efficient, long lifespan, multiple degree‐of‐freedom (multi‐DOF), and high‐performance soft actuators.^[^ ^6^, ^7^ ^]^

Achieving multidimensional motions with large range while maintaining structural stability is essential for soft actuators to meet the diverse demands of practical soft robotics applications. This requirement has been demonstrated in various existing systems, including full‐range joint actuation in wearable rehabilitation devices,^[^ ^8^ ^]^ adaptive grasping of objects with motions of variable curvature, shapes, and sizes in soft manipulators,^[^ ^9^ ^]^ and motion trajectory requirements for omnidirectional locomotion in mobile robots operating in unstructured environments.^[^ ^10^ ^]^ These application scenarios demand actuators capable of large, multidirectional motions to effectively cover the required workspace. In particular, when used in wearable devices and soft grippers, SPAs are expected to achieve both large deformations and structural stability while maintaining compactness. However, soft materials often undergo highly coupled and large‐strain deformations during the movements of the actuator, which induce structural instability and pose significant challenges to the output performance of soft actuators.

The motions of soft actuators are typically achieved through structural designs incorporating asymmetrically distributed constraints, which induce anisotropic stiffness and enable controllable deformations in response to actuation inputs.^[^ ^11^ ^]^ Such motions include elongation,^[^ ^12^ ^]^ contraction,^[^ ^13^ ^]^ bending,^[^ ^14^ ^]^ twisting,^[^ ^15^ ^]^ and multidimensional motions enabled by coupling.^[^ ^16^, ^17^ ^]^ The ability to tailor deformations and mechanical properties by utilizing creases as embedded constraints has made prismatic origami structural designs increasingly popular for developing soft actuators with enhanced motion accuracy and desired stiffness.^[^ ^11^, ^18^, ^19^, ^20^, ^21^ ^]^

The crease pattern, consisting of straight creases or curved creases,^[^ ^22^ ^]^ determines the behavior of the structure. Among these, the straight crease origami (SCO) soft actuator ^[^ ^15^, ^23^, ^24^, ^25^ ^]^ such as the Yoshimura patterned actuators,^[^ ^26^ ^]^ and the curved crease bellows (CCB) soft actuator have emerged as predominant configurations,^[^ ^27^, ^28^, ^29^ ^]^ owing to their predictable deformations and fabrication scalability.^[^ ^15^, ^30^, ^31^, ^32^ ^]^ Prismatic tubes formed by straight creases exhibit excellent axial expandability, but are prone to inward collapse under negative pressure or external disturbances. CCB designs with curved creases and convex cross‐sections offer improved structural stability under similar conditions, but exhibit excessive in‐plane stretching during large‐range motion, leading to increased energy dissipation and material fatigue due to plastic deformations. Prismatic actuators based on straight crease designs have been explored in previous studies, such as the soft origami for gripper,^[^ ^23^ ^]^ the facet buckling actuator utilizing a Tachi–Miura origami pattern,^[^ ^33^ ^]^ and straight‐crease origami structures capable of supporting large loads.^[^ ^34^ ^]^ Similarly, various curved‐crease actuators have been successfully demonstrated in existing studies, including the design of bidirectional bending soft actuators,^[^ ^35^ ^]^ bellows actuators widely used in soft robotics,^[^ ^36^, ^37^, ^38^, ^39^ ^]^ and multi‐stable soft actuators based on curved‐crease origami structures composed of planar and curved facets.^[^ ^40^ ^]^ However, existing origami prismatic designs have yet to emphasize achieving both large range of motions and structural stability. By combining the characteristics of straight and curved creases, a potential pathway is opened for the design of crease patterns on the surfaces of soft prismatic structures, enabling actuators with large‐range motions, desired anisotropic stiffness and enhanced stability.

To achieve diverse motion modes in soft robotics systems, multiple actuation units are adopted and arranged to coordinate the motions.^[^ ^41^, ^42^ ^]^ Researchers proposed modular integration strategies, where coordinated motion is achieved through serial and parallel arrangements of actuation units.^[^ ^32^, ^43^, ^44^, ^45^ ^]^ Liu et al. designed soft robotic flippers by constraining origami actuators using kirigami structures.^[^ ^45^ ^]^ Liu et al. proposed a soft robotic joint with multiple origami actuators capable of twisting and antagonistic motion.^[^ ^43^ ^]^ Li et al. developed a versatile gripper by connecting several soft actuators in series through different configurations.^[^ ^15^ ^]^ These works achieve multidimensional motions by external constraints or modular designs. However, additional connections and constraints hinder the compactness and introduce deformation mismatches between auxiliary components, especially during multidimensional motions.

Recently, multi‐chamber actuators have emerged as a novel solution for generating multi‐DOF motions within one body.^[^ ^18^, ^46^, ^47^ ^]^ This approach involves dividing its internal space into multiple chambers by structural design. By inflating and deflating the inner chambers with diverse actuation modes, antagonistic or synergistic interactions can be generated, enabling multidimensional movements with a compact profile. Zhou et al. developed a bidirectional SPA that uses an Acrylonitrile Butadiene Styrene plate to divide the actuator into two chambers.^[^ ^48^ ^]^ Similarly, Zhu et al. designed a soft actuator composed of two chambers with a flat dividing wall,^[^ ^49^ ^]^ while Bell et al. developed a symmetric bidirectional bellows actuator with embedded wire mesh.^[^ ^19^ ^]^ These actuators feature dual‐chamber designs separated by an internal non‐foldable partition wall, hence, most of these actuators achieve contraction or extension with buckling and large‐strain in‐plane stretching on the partition wall.

While enabling actuators to achieve compactness and multidimensional motions, the complicated multi‐chamber structures present significant challenges in fabrication due to the presence of concave and convex features within the confined internal space. Existing fabrication techniques can be broadly categorized into three types: 3D printing, heat sealing, and casting and molding.^[^ ^50^ ^]^ Researchers have used Fused Deposition Modeling 3D printing to fabricate multi‐chamber SPAs by adjusting single‐layer extrusion parameters to form airtight chamber walls.^[^ ^18^ ^]^ Meanwhile, researchers have explored the use of heat sealing techniques to produce lightweight SPAs.^[^ ^51^, ^52^ ^]^ However, these fabrication methods still face limitations including limited options for wall thickness and low repeatability. One‐step casting and molding with a single material has the potential to address these fabrication challenges by enabling efficient, rapid, and precise manufacturing. However, it remains highly challenging to fabricate single‐material SPAs with a prismatic structure and complicated internal features.

To address the challenges in developing SPA with large‐range, multidimensional and stable motions, including the design, fabrication, and modeling for implementation in a series of representative applications, this paper presents the design of a dual‐chamber, one‐step molded soft actuator with a proposed straight‐curved crease prismatic origami structure. The proposed straight‐curved crease origami prismatic (SCOP) actuator integrates SCO facets, which provide axial rigidity, with CCB facets that offer high bending compliance, and strategically embeds a foldable internal partition. This synergistic structural configuration achieves tailored anisotropic stiffness and enhanced structural stability. A key design feature is the embedded foldable internal partition, which divides the actuator into two chambers and deforms in coordination with the outer origami wall. This design enables multiple actuation modes through selective pressurization of the chambers, all within a compact and monolithic structure. To highlight the advantages of the proposed actuator, a performance comparison with representative state‐of‐the‐art actuators is summarized in **Table** **1**, focusing on elongation/contraction ratio, bending angle, actuation force, and other relevant metrics.

**Table 1:** Design‐performance comparison between the proposed SCOP actuator and representative state‐of‐the‐art multi‐chamber soft pneumatic actuators.

	Ref. [30]	Ref. [56]	Ref. [57]	Ref. [58]	Ref. [20]	Ref. [18]	SCOP
Number of chambers	2	3	3	2	3	3	2
DOF	1	2	2	1	3	2	2
DOF/Chamber number	0.5	0.67	0.67	0.5	1	0.67	1
Bending angle (degree)	90	180	120	106.6	94.7	90	160
Initial length (mm)	162	–	105	60	97	68	90
Elongation length (mm)	–	–	25	–	25	15	46
Elongation percentage	–	–	23.8%	–	26%	23.5%	51.1%
Contraction length (mm)	–	–	–	–	48.5	27.5	50
Contraction percentage	–	–	–	–	50%	40.4%	55.6%
Lateral actuation force (N in 30 kPa)	0.9	–	–	0.3	2.3		1.5
Pneumatic actuation type	Positive	Positive	Positive	Positive	Positive & Negative	Positive & Negative	Positive & Negative

The main contributions of this paper can be summarized as follows:

1. A straight‐curved crease dual‐chamber prismatic design is proposed for the SPA to realize large‐range motions while achieving superior structural stability. The adoption of straight and curved origami creases achieves the specific anisotropic lateral stiffness that contributes to the structural reinforcement while generating 2 degrees‐of‐freedom (DOF) motions. A foldable partition divides the cavity and simultaneously serves as structural reinforcement.
2. The SCOP design enables one‐step fabrication of the dual‐chamber prismatic structure through a molding and casting process using a single material. A wall thickness–airtightness failure risk function is derived to guide the manufacturing process and ensure airtightness. SCOP actuator prototypes are subsequently produced.
3. To analyze the deformation characteristics and coupled motion of the SCOP actuator, nonlinear mechanical and kinematic models are developed and validated by experimental results. These models present the multidimensional motions and establish the relationships among axial force, lateral tip force, and input pneumatic pressure. The effectiveness of the structural reinforcement is further verified through the finite element method (FEM).
4. A versatile wearable robot for assisting thumb and wrist movements is developed to validate the versatility and feasibility of the SCOP actuator's large range of motions and stable performance with multiple actuation modes. The approach's effectiveness and practicality on soft robotics are further verified by the gripper with multimodal gripping and the planar omnidirectional crawling robot, indicating the promising potential.

The rest of this article is organized as follows. Section 2 presents the concept and design of the SCOP actuator, establishes analytical models, and performs simulations and manufacturing. Section 3 conducts characterization experiments and model verification. Demonstrations are presented in Section 4. Section 5 concludes this article.

## 2 Results

### 2.1 Concept and Design of SCOP Actuator

**Figure 1:**
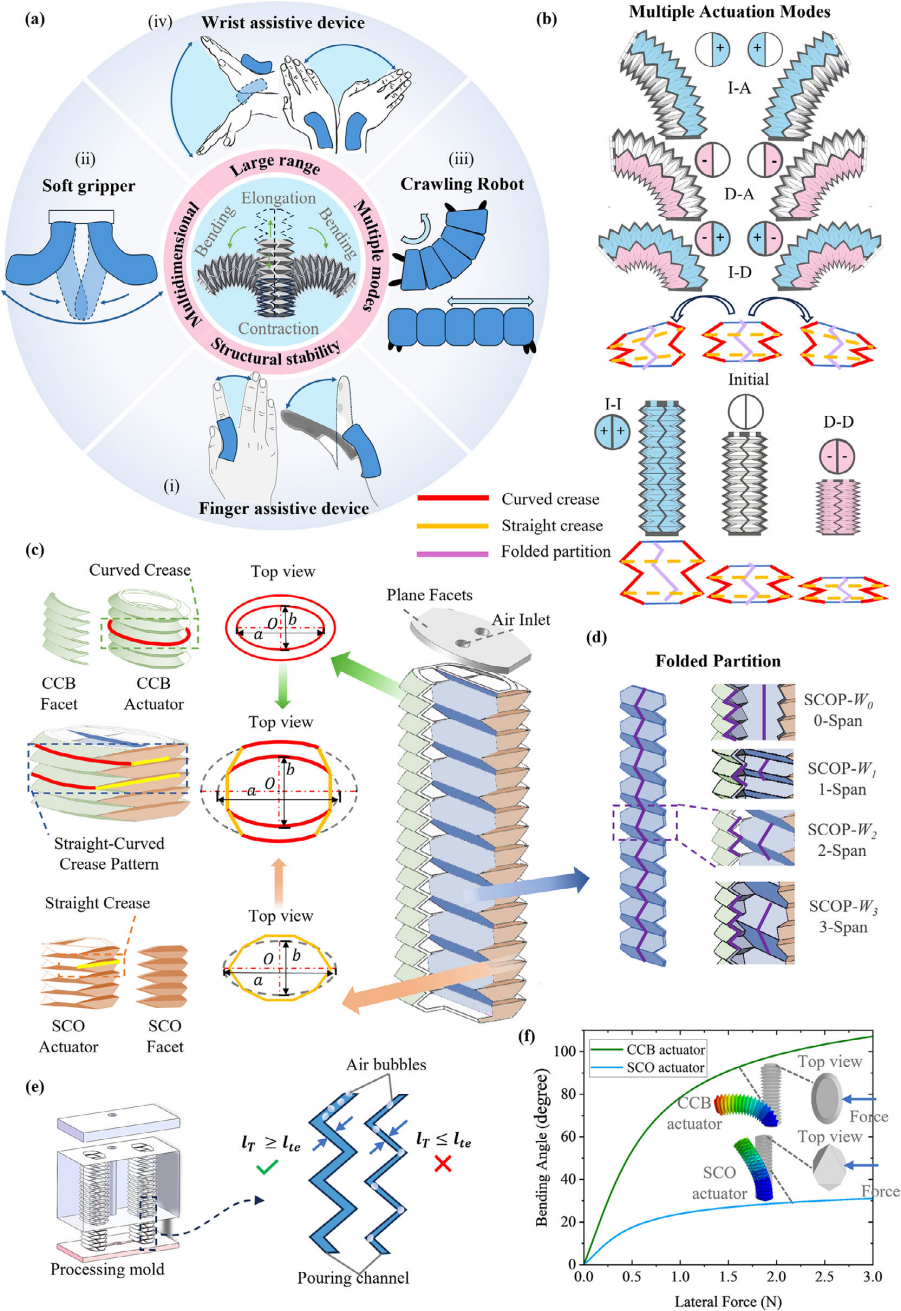
The concept of the SCOP actuator. a) The need for soft actuator with large range of motions, multidimensional motions, and structural stability, in applications including soft wearable robots for (i) assisting finger and (iv) wrist movements, (ii) the two‐finger soft gripper, (iii) the single‐actuator crawling soft robot. b) SCOP actuator obtains elongation, contraction and bidirectional bending motions. c) Outer wall structural concept consisting of the straight‐curved crease pattern. d) The folded partition is embedded in the structure to construct a soft actuator with a dual chamber. e) The concept of an airtightness risk function is proposed for one‐step manufacturing. f) Comparison of the bending characteristics of CCB actuator and SCO actuator through FEM.

Soft robotic applications require actuators capable of large‐range and multidimensional motions to effectively cover the intended workspace, as shown in **Figure** **1**a. Interactive scenarios such as wearable devices (i and iv), soft grippers (ii), and adhesion‐based crawling (iii) often demand surface conformity to ensure purposeful interaction (Figure 1a). These scenarios require actuators to simultaneously achieve stretchability and flexibility while ensuring large‐range motions, structural stability, and compactness through the structural design.

Aiming for such requirements, we propose the prismatic dual‐chamber soft actuator design, with purposefully and compactly distributed straight and curved origami creases to pattern the outer walls (Figure 1c), and an embedded foldable partition (Figure 1d) to divide the internal space into two chambers. 3D‐printed nylon planar facets are used to seal both ends of the prismatic structure for airtightness, with two air inlets integrated into one end for connection to the pneumatic system.

To accumulate folding motions for the output of the actuator, the identical foldable layers are stacked along the axis direction of the structure. By increasing the number of layers (*N_a_*), the range of motion can be directly extended, while the elongated tubular volume sabotages the stability of the structure during the pneumatic actuation. To simultaneously obtain large‐range motions and superior structural stability, the external walls of the SCOP soft actuator are designed with CCB facets and SCO facets, defined by the curved and straight creases, respectively. As shown in Figure 1c, the CCB facet and the SCO facet are symmetrically distributed on opposite surfaces, with the CCB facets being wider than the SCO facets. The cross‐section can be considered as an elliptical prismatic shape, whose major axis is perpendicular to the straight creases and the minor axis is perpendicular to the curved creases. The straight facets connect the CCB facets and serve as a supporting structure to improve buckling resistance and enhance stability.

To generate multidimensional motions, independent chambers are formed inside the actuator by embedding partition walls as shown in Figure 1d. Due to a lack of deployability, the non‐folding partition design constrains axial DOF of the actuator and is therefore unable to achieve the desired motions. Therefore, a folded partition is embedded within the structure serving as a shared partition between the two chambers and effectively dividing the actuator into two cavities. The foldable partition along with the outer wall allows the structure to elongate, contract, and bend with specific accumulations of folding deformations at the creases. To achieve a larger bending angle, the actuator is designed to bend around curved creases. Thus, the folded partition is oriented perpendicular to the SCO facets. The folded partition can be customized with varying numbers of layers, provided it connects to and aligns with the creases of the SCO facets. The folded partitions are characterized by *W_i_* ​​​ (*i* = 0, 1, 2, 3). The relationship between the folded partition and the outer wall is described by the *i*‐span, where *i* denotes the number of outer wall folding layers spanned by each folded partition layer. For instance, the *W_1_* folded partition spans one outer wall layer per folding layer, corresponding to a 1‐span configuration. As shown in Figure 1d, the structural designs of the partition walls are presented for four configurations, where *i* = 0, 1, 2, 3.

To verify the choice of bending direction, a FEM simulation is used to show the range of bending. The CCB‐only and SCO‐only soft actuators as listed in Table S1 (Supporting Information) are compared, which share the same height *H*_0_, the ratio between the length of the major axis (*a*) and the minor axis (*b*), dihedral angle and number of foldable layers, as shown in Figure 1f. A lateral force is applied to the top end of the soft actuators, and the differences in bending angles are observed. Subjected to a 3 N force, the CCB actuator bends to 107°, while the SCO actuator bends to 31°. The results show that the CCB structure exhibits greater bending compliance than SCO, verifying the design of SCOP to bend along the CCB facets.

To ensure the airtightness of the SCOP actuators produced by the one‐step manufacturing process, an airtightness risk model was developed based on the wall thickness of the SCOP structure. As shown in Figure 1e, the wall thickness corresponds to the width of the pouring channel during fabrication, denoted as *l_T_*. The model defines a critical threshold *l_te_*, which serves as a design guideline to minimize potential leakage points after fabrication. This approach ensures improved quality and repeatability of the SCOP actuators.

Benefiting from the SCOP design, the soft actuator is capable of performing extension, contraction, and bidirectional bending under combinations of positive and negative pressures. As shown in Figure 1b, the actuation mode marked in pink represents negative pressure, blue indicates positive pressure, and white denotes atmospheric pressure. By modulating the inflation and deflation combinations of the two chambers, three variable curvature bidirectional bending actuation modes can be achieved through single‐chamber inflation (I) with the other in atmospheric (A) pressure (I–A), single‐chamber deflation (D) with the other in atmospheric pressure (D–A), and the one chamber inflation and the other deflation (I–D), as shown in Figure 1b. Extension and contraction motions are generated when both chambers are simultaneously pressurized (I–I) or depressurized (D–D), respectively. The SCOP design enables the simultaneous realization of large range of motions, multiple actuation modes, and structural stability, all integrated into a single soft actuator.

### 2.2 Modeling of SCOP actuator

The SCOP actuator's motions and forces output are generated by the deformations of the structure brought by the pressure variations in the two chambers. To control the output, it is crucial to establish correlations among the actuator's displacement, output forces, and dual‐chamber pressures.

**Figure 2:**
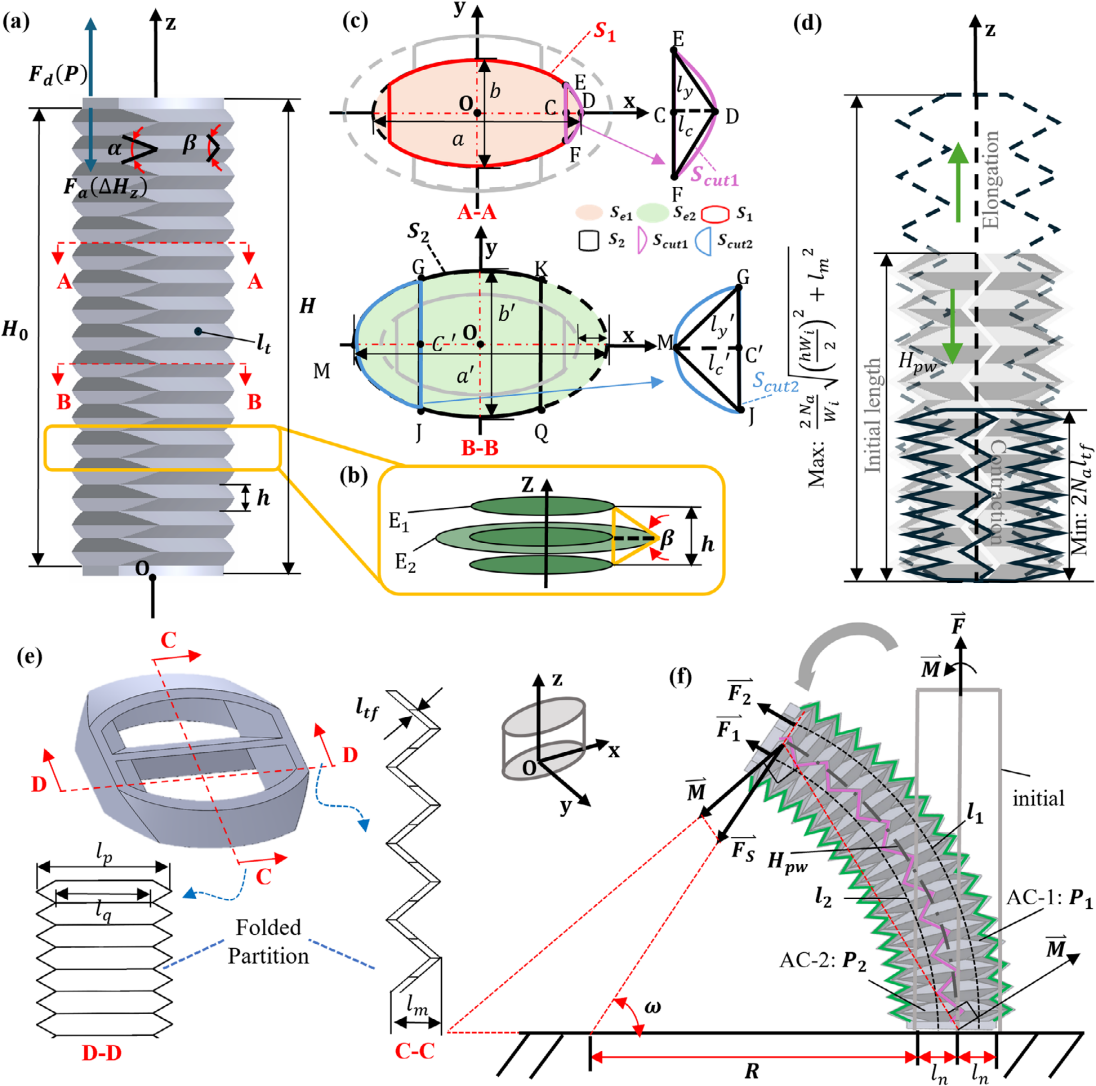
Parameters of the SCOP actuator. a) SCOP actuator outer walls parameters. b) Geometric relationship parameters between cross‐sections. c) A–A section and B–B section and the relationship between them and parameters of ellipse E_1_ and E_2_. d) SCOP's theoretical linear range of motions (elongation and contraction). e) C–C section and D–D section parameters. f) SCOP actuator bending parameters, torque and mechanical parameters of actuator during bending.

The effective cross‐sectional area *S_a_* ​ is a key parameter in deriving the actuator's output force, displacement, and bending angle. According to the geometric relationship between A‐A and B‐B (**Figures** **2**a‐c), *S_a_* can be described as

(1)

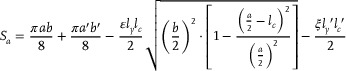

where *a* and *b* are the major and minor axes of ellipse E_1_, and *a′* and *b′* are those of ellipse E_2_. The parameters ε and ξ represent the area ratios of triangles DEF and GMJ within regions CEDF and GMJC′, respectively. *l_c_* and *l_c_'* are the lengths of the line segments CD and MC′, and *l_y_* and *l_y_'* are the lengths of the line segments EC and GC′, respectively, as shown in Figure 2c. Detailed derivation of the effective cross‐sectional area is provided in Supplementary Information Note S1 (Supporting Information).

Based on *S_a_* ​, the statics models focus on the axial linear motion of the actuator can be obtained, which establish the relationship among input pressure, output force, and displacement. The axial output force *F* can be expressed by

(2)

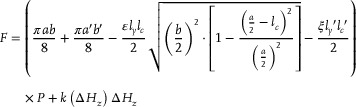

where *F* is the output force of the actuator during axial motion, *P* is the internal pressure, *k*(Δ*H_z_*) is the axial stiffness of the actuator during displacement Δ*H_z_*.

Then, the relationship between the actuator's linear displacement and the variation in internal pressure can be derived as follows:

(3) $$\Delta H_{z}=\frac{S_{a}}{k\left(\Delta H_{z}\right)}\cdot P$$

 For a detailed derivation of the linear motions, please refer to Supplementary Information Note S2 (Supporting Information).

To describe the bending behaviors of the SCOP soft actuator, the relationship between the two chamber pressures (*P*_1_​​ and *P*_2_​) and the resulting bending angle ω is analyzed. The results are derived as

(4) $$\omega =2\sin^{-1}\left(\frac{\left(S_{a}\left(l_{p}+l_{q}\right)\right)}{8H_{0}l_{n}H_{pw}}\cdot \left(\frac{P_{1}}{k\left(\Delta l_{1}\right)}-\frac{P_{2}}{k\left(\Delta l_{2}\right)}\right)\right)$$

where *l_p_* and *l_q_* are the outer and inner widths of the D‐D cross‐section in the folded partition, respectively, as shown in Figure 2e. *H_pw_* is the central arc length of the folded partition. *k* (Δ*l*_1_) and *k* (Δ*l*_2_) are the stiffnesses of the two cavities at axial displacements of Δ*l*_1_ and Δ*l*_2_, respectively, which can be experimentally determined. The parameter *l_n_* denotes the central distance from each of the two chambers to the actuator's central axis, serving as a geometric reference for determining the width of each chamber, as shown in Figure 2f.

The lateral tip force is essential for understanding the actuator's interactive performance. Based on the geometric and static analysis in Figures 2e and 2f, the resulting lateral tip force *F_S_* ​ can be expressed as:

(5) $$F_{S}=\frac{\left(P_{1}-P_{2}\right)\cdot S_{a}\cdot l_{n}\cdot \cos\left(\frac{\omega }{2}\right)}{4\left(H_{0}+\Delta l_{2}+l_{n}\cdot \sin\left(\omega /2\right)\right)}+\frac{6k\left(\Delta H_{z}\right)I\sin\omega }{S_{a}H_{pw}}$$

where the second moment of inertia *I*, corresponding to the SCOP actuator bending along the CCB facets. For a more detailed derivation, please refer to Supplementary Information Note S3 (Supporting Information).

Furthermore, the kinematic model is established, the actuator space [*H_pw_*, ω]is mapped to the task space, obtaining the transformation matrix ***T*** with rotation matrix ***R*** *x* and position matrix ***P*** = [x, y, z]^T^ as follows:

(6) $$T=\left[\begin{array}{cc} R_{x} & P\\ 0 & 1 \end{array}\right]=\left[\begin{array}{cccc} \cos\omega & 0 & \sin\omega & H_{pw}/\omega \left(1-\cos\omega \right)\\ 0 & 1 & 0 & 0\\ -\sin\omega & 0 & \cos\omega & H_{pw}/\omega \sin\omega \\ 0 & 0 & 0 & 1 \end{array}\right]$$

The design of the folded partition influences the actuator's axial extension and contraction range. *H_pw_* has a limited range throughout the folding and unfolding process, which does not involve material stretching or compression. According to the geometric relationship

(7) $$2N_{a}l_{tf}\leq H_{pw}\leq \frac{2N_{a}}{W_{i}}\sqrt{{\left(\frac{hW_{i}}{2}\right)}^{2}+{l_{m}}^{2}}$$

where *l_tf_* is the thickness of the folded partition, *W_i_* is the number of folded layers that each folded partition spans across *i* layer of the outer wall, and *h* is the height of each outer wall layer, as shown in Figure 2d.

### 2.3 Simulations on the SCOP Actuator

To verify the structural stability performance of the SCOP structure under negative pressure, this section presents comparative FEM simulations of three different designs. Specifically, the deformation behaviors of actuators with CCB, SCO, and SCOP designs, with and without the folded partition are investigated given negative input pressure. The FEM analysis purposes focus on validating the following four aspects of the SCOP actuator: 1) the enhancement on structural stability; 2) the folded partition's impact on the axial output, 3) desired lateral stiffness anisotropy, and 4) the impact of the *i‐*span (*i* = 0, 1, 2, 3) of folded partition layers on the axial motion range.

To characterize the actuator's transient collapse behavior, deformation is described by the change in inner cavity diameter. Without a folded partition, collapse occurs when the chamber diameter reaches zero. With a foldable partition, collapse is defined when either chamber's diameter reduces to zero. During the processes, the axial contraction is recorded simultaneously during the process.

**Figure 3:**
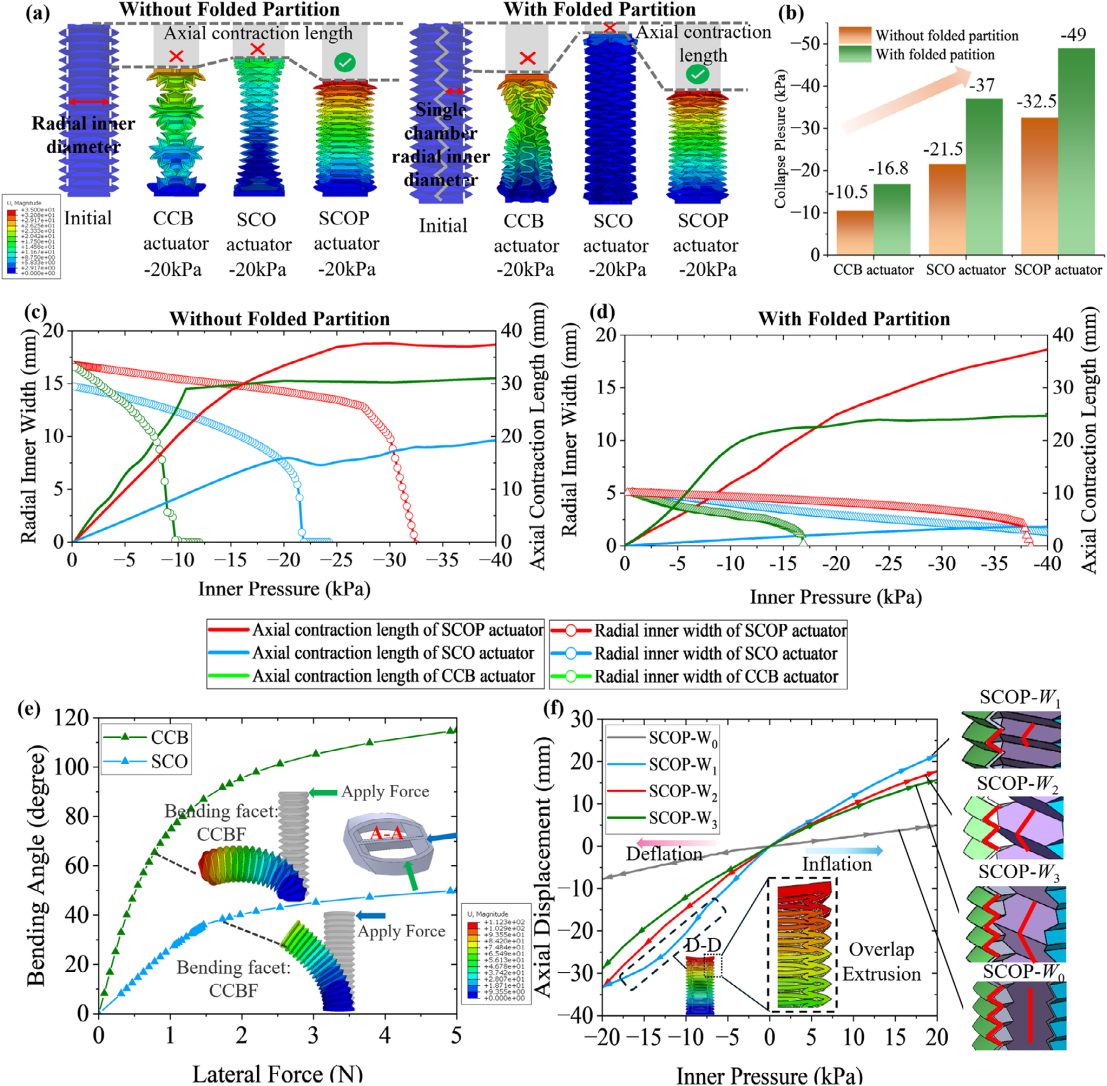
FEM of the SCOP actuator. a) FEM results of CCB actuator, SCO actuator and SCOP actuator with and without folded wall at −20 kPa. b) Collapse pressure of CCB actuator, SCO actuator and SCOP actuator with and without folded partition. c) The axial displacement changes and radial displacement changes of the CCB actuator, SCO actuator and SCOP actuator under negative pressure without folded partition are compared. d) Collapse pressure of CCB actuator, SCO actuator and SCOP actuator with folded partition. e) Comparison of the bending deformation angles of the SCOP actuator along SCO and CCB, when the same force is applied. f) The influence of folded partition spanning W_1_, W_2_ and W_3_ external structural fold layers and non‐folded partition wall on the axial displacement of the actuator.

As shown in **Figure** **3**a, subjected to a negative pressure of −20 kPa, the CCB actuators exhibit obvious collapse regardless of the folded partition's reinforcement. This failure may result from a sudden transition of ridge creases into valleys, which prevents proper folding of the crease pattern. The buckling induces radial collapse of the actuator, thereby impairing its folding behavior and limiting axial output. The SCO actuator exhibited limited axial contraction, which may be attributed to its excessively high axial stiffness. In comparison, the SCOP actuator does not collapse under a negative pressure of −20 kPa while outputting stable contraction, regardless of whether the folded partition is integrated. These results preliminarily confirm the effectiveness of the SCOP structure in resisting buckling under negative pneumatic input.

To further evaluate the designs under negative pressure, the pressure ranging from 0 to −40 kPa was applied to the actuators. Axial contraction and inner diameter were recorded throughout the process. As shown in Figure 3c,d, without the folded partition structure, the CCB actuator collapsed at −10.5 kPa, the SCO at −21.5 kPa, while the SCOP maintained stability until −32.5 kPa, as shown in Figure 3c. The SCOP actuator exhibited a 209% improvement in collapse resistance over the CCB, and a 51% improvement over the SCO (Figure 3b). With the folded partition, the collapse resistances were significantly enhanced for all three types, namely, the CCB actuator collapsed at −16.8 kPa, the SCO at −37 kPa, and the SCOP at −49 kPa, as shown in Figure 3d. These results show improvements of ≈60%, 72%, and 50% respectively compared to the corresponding single‐chamber designs. By incorporating the folded partition, the SCOP actuator achieved significantly higher collapse pressure, exceeding that of the CCB and SCO by 192% and 32%, respectively, as shown in Figure 3b.

When collapse occurred, the slope of the axial displacement curve shows a sharp decrease (Figure 3c,d), indicating that structural instability significantly degrades the actuation performance. The addition of the folded partition shows no effect on the axial contraction behavior of the SCOP actuator, demonstrating the deformation compatibility between the folded partition and the outer walls. Therefore, SCOP design enhances the actuator's stability under negative pressure and simultaneously maintains the axial displacement performance, validating the advantages as the structural design of soft actuators.

The bending angles around the CCB facets and SCO facets are compared using the SCOP actuator in simulations. As illustrated in Figure 3e, a lateral force was applied to the actuator's top end, and the resultant bending angle was measured. Simulation results show that under a 5 N lateral load, the bending around the CCB obtained a rotational angle of ≈110°, whereas the bending around the SCO only reached ≈50°. These results validate the effectiveness of the anisotropic stiffness design of the SCOP actuator, which collectively enhances structural stability while enabling large range of rotational motions.

To investigate the effect of the folded partition's indexed‐layer folded design on the axial displacement performance of the actuator, FEM simulations were performed on SCOP actuators with different foldable partition designs, i.e., SCOP‐*W_i_*, *i* = 0, 1, 2, 3, where *W_i_* is the number of folded layers that each folded partition span *i* layers across the outer wall. The chambers were applied pressures ranging from −20 to 20 kPa, with the corresponding axial displacements recorded as shown in Figure 3f. The results show that SCOP‐*W_0_* exhibited significantly lower axial displacement compared to the actuators with foldable partitions. This can be attributed to the lack of foldability of the *W_0_* partition, which fails to provide sufficient axial compliance during actuation. Instead, motion relies primarily on the in‐plane stretching deformation of the *W_0_*, leading to restricted displacement output. SCOP‐ *W_1_* demonstrated the largest axial displacement output among the four designs. This is due to the compatible folding motions between the *W_1_* and the outer walls which share the same number of layers, enabling high compliance during axial motion. However, a sudden change in slope (i.e., an increase in stiffness) was observed near −10 kPa, suggesting a nonlinear stiffening behavior. Simulation results indicate that excessive folding leads to contact between adjacent layers under negative pressure, thereby excessively increasing structural stiffness and constraining displacement output. Both SCOP‐*W_2_* and SCOP‐*W_3_* exhibited stable axial displacement, with SCOP‐*W_2_* showing a slightly larger displacement range than SCOP‐*W_3_*. Therefore, the configuration of SCOP‐*W_2_* offers better coordination between the folded partition and outer wall, enabling an effective balance between large‐range motions and stable output. As a result, SCOP‐*W_2_* was selected for further investigation in this study.

### 2.4 Fabrication of SCOP Actuator

To achieve large‐range motion in the slender dual‐chamber structure with straight‐curved creases, the actuator needs to be fabricated from a single material that provides high deformation compliance between the foldable features and airtightness. To achieve cost‐efficient, accurate and repeatable manufacturing for stable performance of the actuator, a one‐step molding process is in favor. To enable one‐step molding fabrication on the dual‐chamber body, a commercially available polyurethane‐based silicone rubber, Hei‐Cast 8400, was selected due to its excellent elasticity and compatibility with cost‐effective industrial casting techniques. The actuators were fabricated through a molding and casting process, allowing efficient one‐step production using a single material.

**Figure 4:**
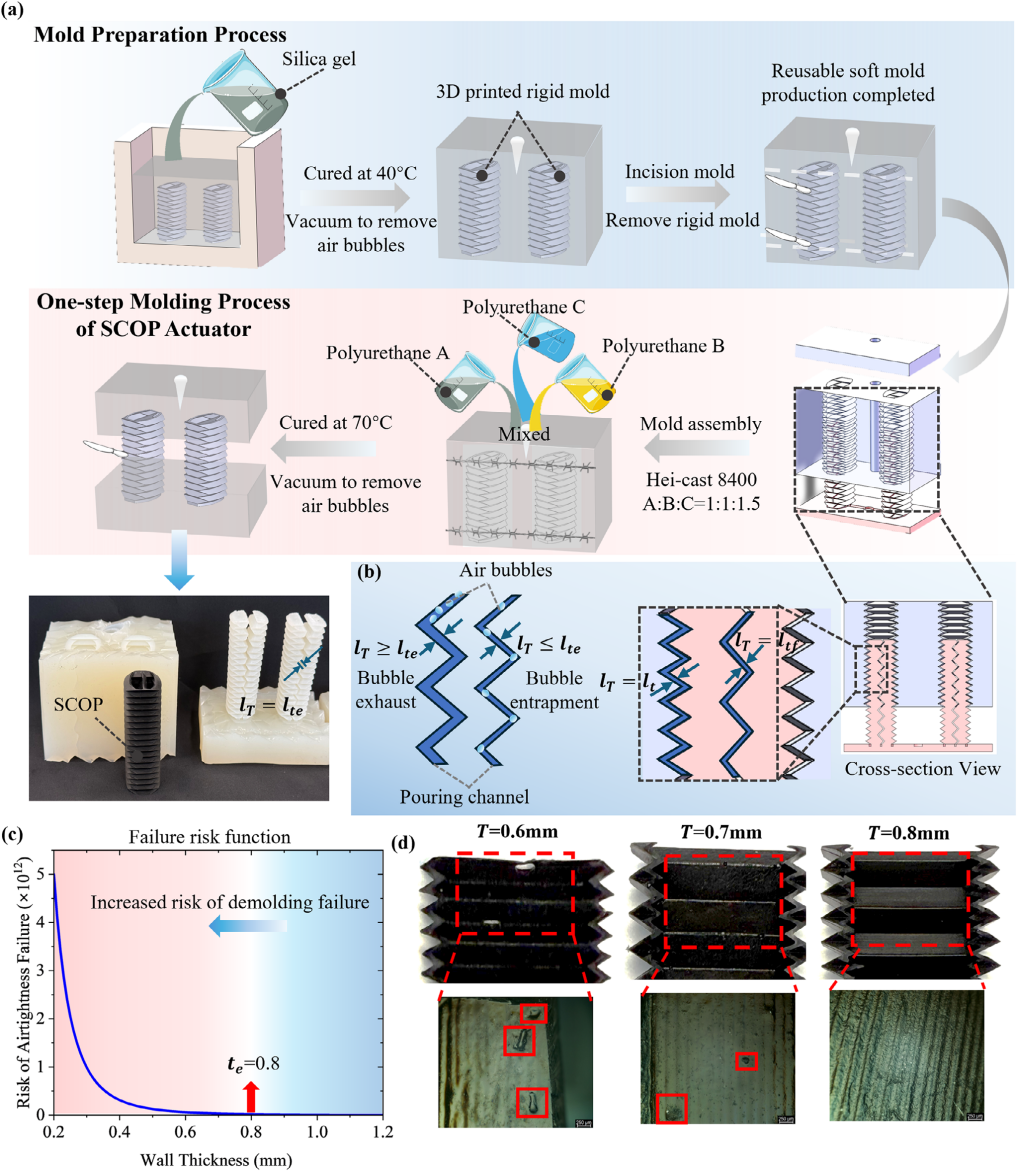
Fabrication process of the SCOP. a) SCOP manufacturing process flowchart includes the mold preparation process and the one‐step molding process. b) Conceptual diagram illustrating how the wall thickness defines the pouring channels, and how the channel width affects air bubble removal during molding. c) The result of a wall thickness–airtightness failure risk function. d) The surface characteristics of SCOP actuators with different wall thicknesses were compared under a microscope, focusing on the same top of actuator regions along the D–D cross‐section.

First, as shown in **Figure** **4**a, the fabrication includes two main processes: mold preparation and one‐step molding. Silicone (Semitransparent silicone, No.918, Minghe) needs to be prepared as the base material for the molding process, and this soft mold can be reused. The 3D printed rigid mold (resinous material, 8100, Wenext) is placed in silicone for fabrication of the soft mold. Then, the silicone after vacuum is placed in a 40 °C environment for curing, which produces the soft mold for subsequent operations. After curing, the rigid mold is removed by incising the soft mold. The incision is subsequently re‐stitched and closed, with a small hole open at the top to enable the later injection.

Next is the one‐step molding process of the SCOP actuator. The Hei‐Cast 8400 consists of three components: polyol A, isocyanate B, and polyol C, which are mixed in a ratio of 1:1:1.5 to achieve a Shore hardness of 60. After mixing, the material is injected into the soft mold through a small inlet and then placed in a vacuum machine (CHX600, ‐100 kPa, 50–200 °C, Changxing) for curing at 70 °C for 45 min. If the air bubbles generated during the injection of mixed materials are not properly eliminated at this step, they may form leakage points after curing. Finally, the soft mold is reopened, and the SCOP actuator is demolded, requiring no post treatments. The soft SCOP is sealed with end panels to form the actuator prototype. The detailed one‐step molding process was recorded in Supplementary Movie S1 (Supporting Information).

For the pneumatic soft actuator, ensuring airtightness is a primary concern. The airtightness is closely related to the thickness of the actuator wall, which is determined by the fluid characteristics of the material before curing, due to the pouring process during the molding. During the fabrication process, the uncured mixture is injected into the mold, and the wall thickness effectively defines the flow channels within the mold. For the uncured fluid materials such as Hei‐Cast 8400, which has a viscosity of ≈500 mPa·s at 25 °C (Shore hardness 60), the insufficient wall thickness can result in an incomplete filling of the mold, especially around the crease vertices and at the channel outlets. This leads to stranded air bubbles and leakage points after curing. To investigate the design boundary of wall thickness associated with the fluid characteristics of the material, an airtight failure function was developed based on analytical modeling and previous experience.^[^ ^53^ ^]^ This function serves as a guideline for determining the appropriate wall thickness. For qualitative analysis, combining the Laplace pressure formula and Hagen–Poiseuille law (laminar flow) yields^[^ ^54^ ^]^

(8) $$\Delta P_{\mathrm{Laplace}\;}=\frac{2\gamma }{l_{T}}$$

(9) $$Q=\frac{\pi {l_{T}}^{4}\Delta P}{8\mu L}$$

where γ is the surface tension between the material and air, and *l_T_* is the width of the wall thickness of the SCOP. Equation (8) presents the relationship between the wall thickness and the ability of bubbles to be effectively evacuated. From the Hagen–Poiseuille law (laminar flow),^[^ ^55^ ^]^ it can be deduced that *Q*∝*l_T_* ^4^. When the channel width is excessively small, the material may fail to completely fill the crease vertices, resulting in a failure of airtightness.

As shown in Figure 4b, the outer chamber wall thickness is represented as *l_T_* = *l_t_* , while for the folded partition thickness, it is denoted as *l_T_* = *l_tf_* . Combining the Eqnuation (8) and Equation (9), we established the airtight failure function:

(10) $$R_{\mathrm{fail}}\left(l_{T}\right)=\alpha \cdot \frac{1}{l_{T}}+\beta \cdot \frac{1}{{l_{T}}^{4}}$$

where α and β are empirically given as α  =  3 and β  =  5 × 10^−4^, derived through calculation based on the existing study.^[^ ^53^ ^]^ The trend of the failure function is depicted in Figure 4c. The critical value of *l_T_* is determined to be ≈0.8 mm. When the wall thickness is less than 0.8 mm, the risk of actuator failure increases sharply.

To validate the effectiveness of the failure function, SCOP actuators with three different wall thicknesses (*l_T_* = 0.6, 0.7, and 0.8 mm) were fabricated. Under digital microscope (DVM6, Leica), surface characteristics were examined at the same region, with particular attention to trace residual air bubbles. As shown in Figure 4d, the actuator with a thickness of 0.8 mm exhibited a clear and smooth surface texture, whereas those with thicknesses of 0.6 and 0.7 mm showed numerous porous marks generated by the remaining bubbles. These observations indicate that a thickness of 0.8 mm achieves the complete release of air bubbles during fabrication, while thinner walls tend to trap more bubbles, most of which accumulate near the casting outlet (i.e., the top of the actuator), significantly increasing the risk of airtightness failure.

The proposed SCOP actuator was fabricated for experimental characterization, and its design parameters were determined by combining the results with the FEM simulations and fabrication requirements, as listed in Note Table S2 (Supporting Information). Supplementary Information.

## 3 Model Validation and Characteristics of SCOP Soft Actuator

To validate the modeling and investigate the mechanical properties and motion characteristics of the SCOP actuator, a series of experiments were carried out.

### 3.1 Experimental Setups

**Figure 5:**
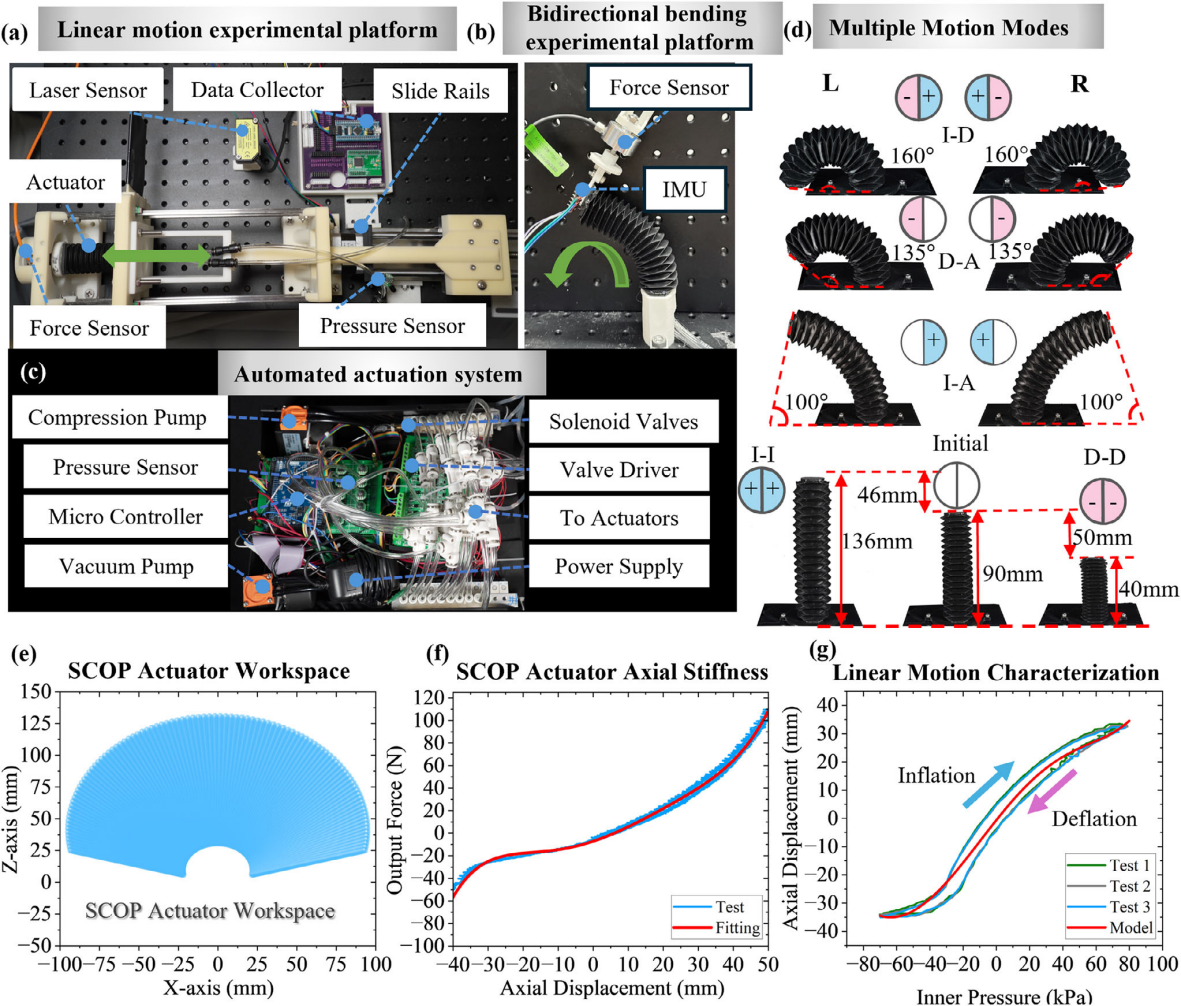
Characteristics and experimental verification of the SCOP actuator. a) Linear experimental setup for testing characteristics of the SCOP actuator. b) Bending experimental setup for testing characteristics of the SCOP actuator. c) An actuation system that can provide a stable pressure supply for channels of the SCOP actuator. d) SCOP actuator motion characteristics under different actuation modes. e) The theoretical workspace of the SCOP actuator. f) The relationship between the output force and displacement of the SCOP actuator in the axial direction under no air pressure input. g) The relationship between linear displacement and pressure during inflation and deflation.

To meet the requirements of multidirectional motion testing, the linear motion and bidirectional bending experimental platforms were developed. **Figure** **5**a shows the linear motion platform and Figure 5b presents the bidirectional bending experiment platform. A pneumatic actuation system is developed to provide the air source and is connected to the experimental device, as shown in Figure 5c. More details of the experimental setup are given in Note S7 (Supporting Information) of the Supplementary Materials.

### 3.2 Actuator Characterization

#### 3.2.1 Large‐Range Motion Characteristics

As shown in Figure 5d, the SCOP actuator is capable of performing multidimensional motions with varying curvatures and a large range of motions by different actuation modes. These motions include extension, contraction, and bidirectional bending. Specifically, the actuator can extend by 46 mm, which corresponds to ≈51.1% of its initial length of 90 mm, and contract by 50 mm, 55.6% of initial length. This results in a total axial deformation range of 96 mm, which is ≈106.7% of its initial length, demonstrating a significant large‐range motion capability. During bidirectional bending, different actuation modes lead to distinctive bending behaviors. The actuator achieves a bending angle of ≈100° under the I–A mode, 135° under the D–A mode, and a maximum of 160° under the I–D mode. This superior bending performance of the I–D mode is attributed to the greater bending moment it generates between two chambers compared to I–A and D–A modes. A design‐performance comparison between the proposed SCOP soft actuator and representative state‐of‐the‐art multi‐chamber soft pneumatic actuators is presented in Table 1. The selected actuators shared characteristics in actuation method, structural design, motion capabilities and application scenarios. The SCOP actuator exhibits superior structural innovation, achieving a DOF‐to‐chamber ratio of 1 and enabling elongation, contraction and bidirectional bending within a single, compact structure. The SCOP actuator demonstrates outstanding performance in key aspects such as elongation, contraction, bending range, and lateral actuation force. Moreover, its ability to operate under both positive and negative pneumatic pressure further enhances its functional versatility and adaptability to diverse soft robotic tasks.

#### 3.2.2 Workspace of the SCOP

Based on the experimentally validated kinematic model Equation (6), the workspace of the actuator is illustrated in Figure 5e. According to the workspace, the actuator is able to contract to a minimum length of 30 mm and extend to a maximum of 135 mm, while achieving bidirectional bending angles of up to ±165°. The model's predictions show good agreement with the experimentally measured maximum extension, contraction, and bidirectional bending, thereby validating the accuracy of the kinematic model.

#### 3.2.3 Axial Stiffness Characteristics

To characterize the axial stiffness behavior, the actuator is tested in the linear motion experimental platform with a chamber connected to atmospheric pressure. In the initial state, one end of the SCOP actuator is connected to a force sensor, while the motorized slide rail drives a 3D‐printed push rod to move slowly and uniformly from −40 to 50 mm. The resulting force and displacement curve is shown in Figure 5f. The results show that the actuator exhibits approximately linear behavior within the displacement range of −10 to 20 mm. Beyond this range, noticeable nonlinearities occur in both opposite directions, with the stiffness increasing significantly as the displacement approaches −40 and 50 mm. This nonlinear behavior is primarily caused by internal structural compression in contraction and material stretching in elongation under large deformation, which results in increased resistance. To better reflect these nonlinear characteristics, curve fitting (red line in Figure 5f) was performed using MATLAB 2023. The fitted curve closely follows the experimental trend and provides a reliable basis for subsequent modeling verification.

#### 3.2.4 Axial Displacement Characteristics

It is worth noting that the nonlinear characteristics of the actuator can be effectively characterized by the nonlinear stiffness. By substituting the fitted stiffness result into Equation (3), the theoretical relationship between axial displacement and internal pressure was obtained. To verify the accuracy of the model and characterize the relationship between internal pressure and axial deformation, both air chambers were connected to a shared air path to ensure identical pressure during actuation. The soft actuator gradually inflated from −70 to 80 kPa and then deflated back to −70 kPa. Axial displacement was recorded in real time using a displacement sensor integrated with the linear test platform, and the pressure variations were monitored using a pressure sensor. The experiment was repeated three times to ensure consistency. The experimental results are shown in Figure 5g. When the internal pressure is near zero, the actuator exhibits a larger axial deformation slope, whereas the slope becomes smaller near ± 80 kPa. This behavior can be attributed to the stiffness increasement once the folded partition exceeds the deployable range during elongation, which constrains further deformation. In the contraction phase, the stacking and contact between the facets of the folded partitions hinder further contraction of the structure. For the same pressure, the axial deformation during deflation is greater than that during inflation. The three repeated experiments demonstrate good consistency. A hysteresis phenomenon is present during the inflation and deflation processes, caused by nonlinearities resulting from surface contact or in‐plane stretching, and the intrinsic nonlinearity of the SCOP material. The Root Mean Square Error (RMSE) between the theoretical prediction and the experimental curves were found to be 1.6 mm during the inflation phase and 2.2 mm during the deflation phase. The slightly higher error during inflation can be attributed to the increased nonlinear deformations and material hysteresis, which are more prominent as the actuator expands. This experimental result is consistent with the relationship Equation (3), which establishes a direct mapping between the actuator's axial deformation and the internal chamber pressure.

#### 3.2.5 Bending Motion Characteristics

**Figure 6:**
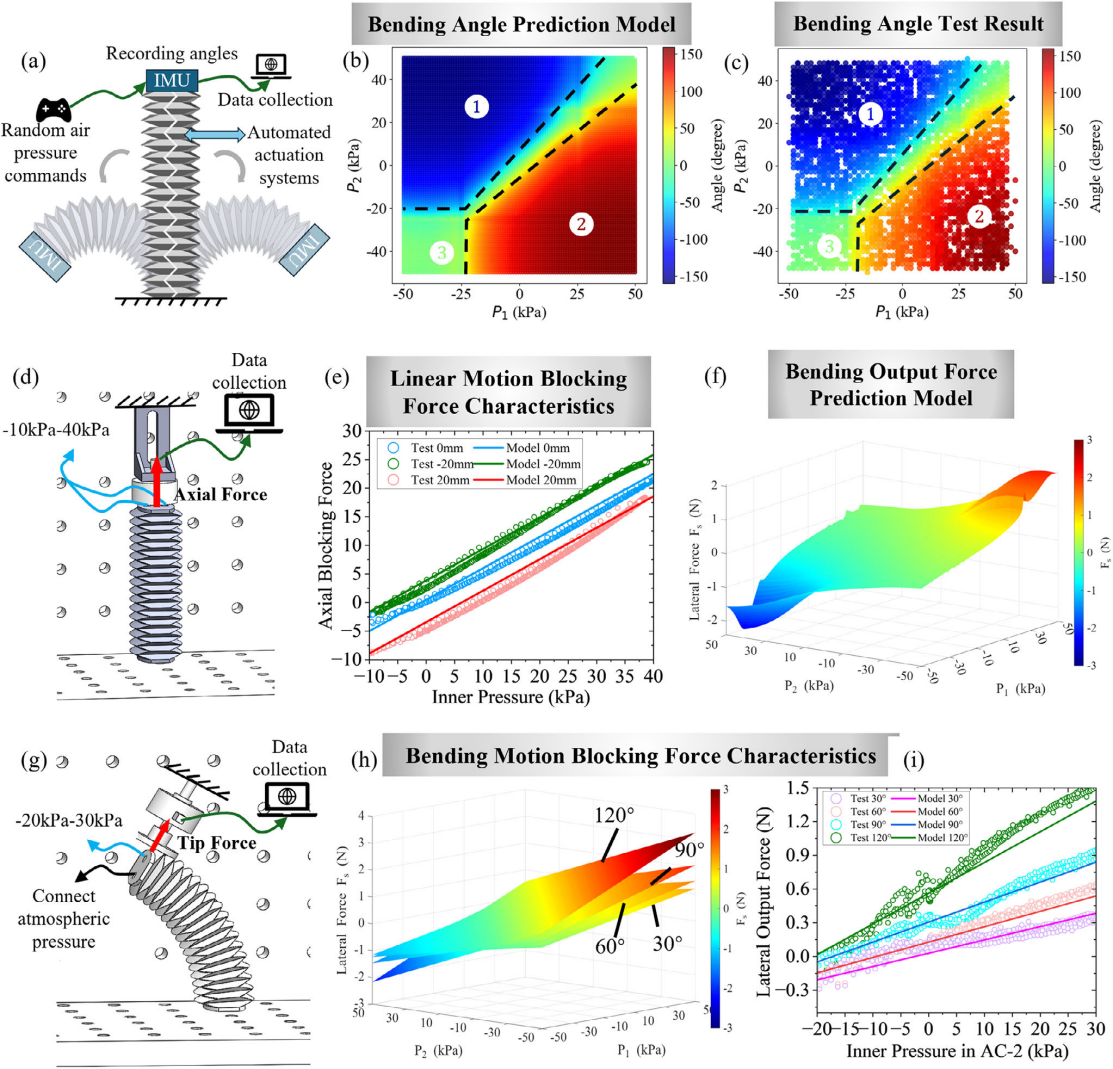
SCOP actuator bending characteristics, model prediction and blocking force. a) Experimental setup for testing the relationship between dual‐chamber pressures and bending angle of the SCOP actuator. b) The model prediction between air pressure in two chambers and the bending angles. c) The relationship between the random combination of air pressure in two chambers and the bending angles and the lifespan of the SCOP with approximately 6000 tests. d) Linear test setup for measuring the blocking force. e) Linear motion blocking force characteristics, including experiments and model comparison. f) The result of prediction model of bending deformation behavior. g) Bending test setup for measuring the blocking force. h) The prediction model of bending block force in different angles. i) Bending motion blocking force characteristics, including experiments and model comparison.

The relationship between the input air pressures and the resultant bending angle is investigated for comprehensively evaluating the actuator's motion capability. The angle prediction model is verified accordingly. The bending motion experiment was conducted using the IMU‐based bidirectional bending experimental platform shown in **Figure** **6**a, in which the two chambers AC‐1 and AC‐2 were independently connected to separate pneumatic channels in the automated actuation system. Each air path was equipped with a pressure sensor to monitor the air pressures, while an IMU was attached to the tip of the actuator to record the bending angle in real time.

To further validate the relationship between the bending angle and the internal pressures in the two chambers (as described in Equation (4)), the theoretical results are plotted in Figure 6b. The pneumatic actuation system was programmed to randomly apply air pressure commands to each chamber, enabling the SCOP actuator to bend in discrete directions with varying angles. The pressure in each chamber was randomly varied between −50 and 50 kPa and held constant for 3 s after each pressurization to allow the actuator to stabilize, ensuring accurate angle measurement.

The experimental results are presented in Figure 6c. It can be observed that when the input pressures are equal (P_1_ = P_2_), the actuator undergoes no bending moment, resulting in a bending angle of zero along the diagonal line. As the pressure difference between P_1_ and P_2_ increases, the bending angle increases accordingly. Within the tested pressure range from −50 to 50 kPa, the actuator achieves a maximum bending angle of ≈150°.

Comparing the theoretical (Figure 6b) with the experimental results (Figure 6c), the bidirectional bending behavior of SCOP can be categorized into 3 regions, as indicated by the dashed boundaries in the figures. Region 1, located in the upper‐left area of the plot, corresponds to conditions where P_2_ > P_1_ ​, indicating the AC‐2 dominates the bending motion. This results in negative bending angles, as the actuator bends toward AC‐1. In contrast, Region 2 (lower‐right) represents scenarios where P_1_ > P_2_ ​; here, the AC‐1 dominates, producing positive bending angles toward AC‐2. Region 3, situated in the lower‐left corner, occurs when both P_1_​ and P_2_​ are negative. Under this balanced contraction, the actuator exhibits minimal or near‐neutral bending, due to the symmetric actuation pressures of both chambers. These regions highlight the actuator's directional response characteristics and serve as a foundation for designing pressure control strategies. Although the experimental data generally align with the theoretical model, discrepancies are observed, particularly near the boundaries between regions. These deviations can be attributed to the inherent hysteresis of soft materials, where the actuator does not fully return to its original shape before the next pressure input is applied.

This experiment was also conducted to evaluate the lifespan of the SCOP actuator. During the process of the experiment, approximately 6000 repeats were performed under various pressure combinations. Throughout this process, the actuator consistently maintained stable output without any structural damage, demonstrating excellent structural stability and long‐term operational reliability. Due to the diversity of pressure combinations applied to the two chambers, the actuator produces a large range of bending movements, thereby demonstrating its multidimensional motion capabilities. Since different pressure combinations of the chambers can result in different bending modes, the theoretical model serves as a valuable reference for designing input control strategies in applications.

#### 3.2.6 Axial Output Force Characteristics

To investigate the axial output force of the SCOP actuator during interaction with the external environment and to validate the theoretical model Equation (2), we measured the blocking force. As shown in Figure 6d, the SCOP actuator was fixed to a linear experimental platform under three different initial axial deformation conditions: compressed by 20 mm, at initial length, and stretched by 20 mm, i.e., ∆H = −20, 0, and 20 mm, respectively. Equal pressures were simultaneously applied to both chambers in each state, gradually increasing from −10 to 40 kPa and then decreasing back to −10 kPa, allowing the pneumatic energy to be fully converted into output force. Throughout the process, the output forces were continuously recorded. The experimental results are shown in Figure 6e, which show good consistency with the theoretical model, indicating that the model can be effectively used to predict the output force when the SCOP actuator is applied for axial actuation.

#### 3.2.7 Lateral Output Force Characteristics

To illustrate the characteristics of the lateral force generated by internal pressure during SCOP bending, the theoretical distribution of *F_b_* (*P*_1_,*P*_2_) was plotted, as shown in Figure 6f. A noticeable nonlinear behavior can be observed, with the maximum output force occurring when the pressure difference between *P*_1_​ and *P*_2_​ is the greatest. This characteristic provides a predictive understanding of the actuator's deformation behavior before arriving at the target contact point, which supports the derivation of tip force during interaction with the environment and offers guidance for controlling bending motion in applications.

In application scenarios, soft actuators are often pre‐constrained at a specific bending angle prior to actuation, in order to guide the direction of motion and force output. Based on this consideration, the end lateral output force (tip force) generated during interaction with the environment was investigated. To validate the theoretical model Equation (5) which are calculated and plotted in Figure 6h, the tip force of the SCOP actuator was measured under various pre‐fixed bending angles. In the experiments, one end of the actuator was fixed, while the other end was pre‐bent and constrained at angles of 30, 60, 90, and 120°. As shown in Figure 6g, a force sensor was positioned perpendicular to the plane of the actuator tip to measure the lateral output force. AC‐1 was left open to the atmosphere, while AC‐2 was connected to the pneumatic system. The input pressure was gradually increased from −20 to 30 kPa, and then decreased back to −20 kPa. Throughout the process, the output force was continuously recorded by the sensor, allowing for the analysis of the relationship between the input pressure and the tip force at different pre‐bent angles. The experimental results are shown in Figure 6i, demonstrating strong agreement with the theoretical predictions. The results show that a larger initial bending angle leads to a greater lateral output force. Additionally, the passive force induced by lateral stiffness in the absence of pneumatic input also increases with the initial bending angle. These findings suggest the potential in applications requiring prestress, such as the wearable assistive devices, where a corrective prestress prior to actuation is need to ensure the initial position.

## 4 Applications in Soft Robotics

To validate the practicality of the SCOP actuator, three typical soft robotic applications utilizing the actuator's large‐range, multidimensional motions and in need of the structural stability are presented in this section: 1) A versatile wearable soft assistant device designed to assist the finger and wrist; 2) A multi‐modes soft gripper; 3) A single‐actuator crawling robot (SCR).

### 4.1 Versatile Soft Wearable Assistive Device

**Figure 7:**
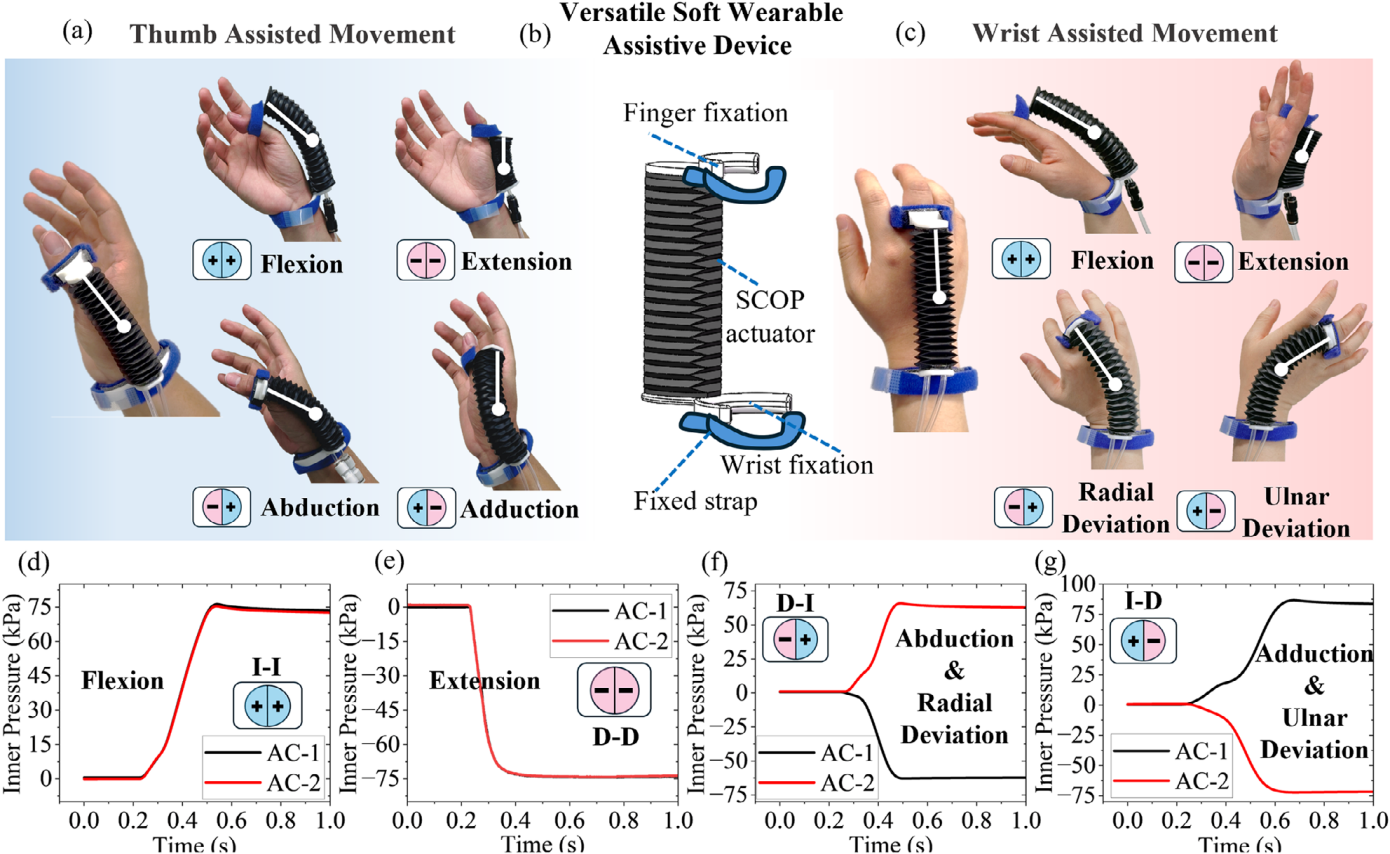
Demonstrations of the soft wearable robot for thumb and wrist. a) A single SCOP actuator can function as a wearable assistive device to support thumb movement. b) The configuration of a soft wearable robot. c) A single SCOP actuator can function as a wearable assistive device to support wrist movement. d) The I–I actuation mode enables controlled flexion of both the thumb and the wrist. e) The D–D actuation mode enables controlled extension of both the thumb and the wrist. f) The D–I actuation mode enables controlled abduction of the thumb and radial deviation of the wrist. g) The I–D actuation mode enables controlled adduction of the thumb and ulnar deviation of wrist.

Benefiting from its large‐range and multidimensional motion, the SCOP actuator can function as a universal stand‐alone module for assisting anatomical joints such as the finger and wrist (**Figure** **7**a,c). By combining different actuation modes, it enables full‐range joint motions with stable output, without relying on complex and bulky configurations.

As shown in Figure 7b, the soft wearable robot integrates the SCOP actuator using 3D‐printed fixtures and adjustable straps for secure attachment and effective force transmission. For thumb assistance, the actuator was mounted over the opponents pollicis muscle (Figure 7a), while for wrist assistance, it was placed on the dorsum of the hand and connected to the middle finger to drive wrist motion (Figure 7c).

According to the typical range of motions in healthy adults, the thumb metacarpophalangeal joint requires ≈0–50° of angular motion,^[^ ^59^ ^]^ while wrist movement typically involves a range of ≈0–60°.^[^ ^60^ ^]^ As shown in Figure 7d–g, various actuation modes were tested to evaluate the actuator's ability to drive different joints. As shown in Figure 7a, different combinations of actuation modes were applied to achieve the thumb's range of motions. The I–I and D–D modes induced thumb flexion and extension, respectively, each achieving a bending angle of 30°. The I–D mode produced thumb adduction with an angle of 50°, and by reversing the pressure polarity (i.e., inflating AC‐2 and deflating AC‐1), thumb abduction was achieved with a bending angle of 20°. These results demonstrate that the SCOP actuator can fully cover the functional range of the thumb through appropriate combinations of actuation modes.

Importantly, the same actuator and actuation strategies can be directly applied to the wrist joint by simply changing the wearing position, as shown in Figure 7c. Despite the wrist having a broader motion requirement, the SCOP actuator still provides sufficient flexibility and large range of motions. Specifically, the I–I mode induced 50° of wrist flexion, the D–D mode achieved 60° of extension, the I–D mode generated 60° of ulnar deviation, and by reversing the pressure polarity, 30° of radial deviation was achieved. These results demonstrate that the SCOP actuator can effectively drive both the thumb and wrist joints fully and meeting the motion‐range requirements.

All motion tests were conducted on a healthy subject, and the entire process was recorded in the Supplementary Movie S2 (Supporting Information). The results validate the SCOP actuator's adaptability and performance advantages in wearable assistive devices. Its compact design enables seamless transition between joints, further enhancing its application potential in versatile wearable robotic systems.

### 4.2 Multimodal Two‐Finger Soft Gripper

**Figure 8:**
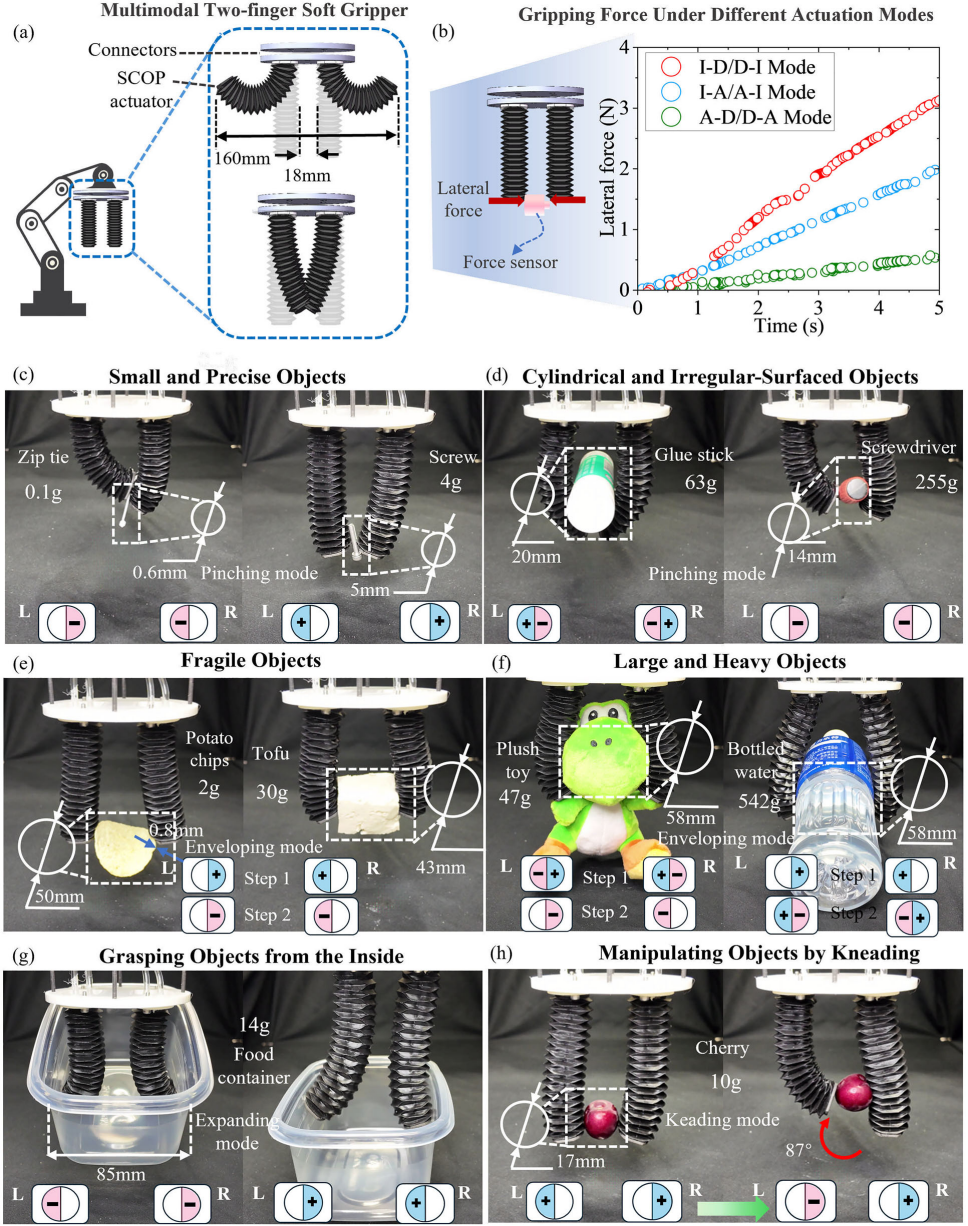
Demonstrations of the two‐finger soft gripper with multi‐modal gripping. a) The configuration of soft gripper and range of operating. b) Grasping force experiments under three actuation mode combinations: I–D/D–I, I–A/A–I, and A–D/D–A. c) Grasping small and precise objects (a zip tie and a screw). d) Grasping cylindrical and irregular‐surfaced objects (a glue stick and a screwdriver). e) Grasping fragile objects (a potato chip and tofu). f) Grasping large and heavy objects (a plush toy and a bottled water). g) Grasping objects from the inside. h) Rotate the cherry 87° by kneading.

By applying different pressure combinations to the SCOP actuator's two chambers, multidimensional and bidirectional bending with distinct curvatures can be achieved. These capabilities allow a SCOP‐based soft gripper to perform various grasping modes for objects of different sizes and shapes. Its inherent compliance supports both active manipulation and passive adaptation. A two‐fingered gripper, using one SCOP actuator per finger, was developed and mounted as the end‐effector of a robot arm (**Figure** **8**a).

To evaluate grasping performance under different actuation modes, force measurements were conducted using two fingers in three actuation modes: I–D/D–I, I–A/A–I, and A–D/D–A (Figure 8b). The I–D/D‐I mode produced the highest force of 3.2 N (D: –80 kPa, I: 50 kPa), followed by 2 N for I–A/A–I (I: 50 kPa), and 0.6 N for A–D/D–A (D: –80 kPa). These results inform the selection of suitable actuation strategies for manipulation tasks.

Based on the combination of the five actuation modes (I–D, I–A, D–A, I–I, D–D), the SCOP‐based soft robotic gripper achieves a variety of grasping strategies to accommodate objects of different sizes, shapes, weights, and features. These grasping modes include inward close pinching (pinching mode), where the fingertips bend toward each other to directly grasp small or slender objects (diameter ≤18 mm), as shown in Figure 8c; outward enveloping (enveloping mode), in which the fingertips first extend outward to envelop larger objects (diameter >18 mm) before closing around them, as shown in Figure 8f; and internally expanding grasps (expanding mode), where the gripper inserts its fingers into hollow or ring‐shaped objects and then extends outward to pick up, as shown in Figure 8g. The gripper is capable of performing twisting or kneading‐like (kneading mode) manipulations by generating opposite bending motions at the fingertips, allowing certain objects to move vertically and rotate between the two fingers, as shown in Figure 8h.

To validate the dexterity and adaptability of the soft gripper in handling objects with different sizes, shapes, surface properties, and levels of fragility, a series of representative objects was selected, as shown in Figure 8c–h. The objects include a fine zip tie (diameter: 0.6 mm, 0.1 g) and an M5 screw (4 g) shown in Figure 8c; as well as a glue stick with a smooth cylindrical surface (diameter: 20 mm, 63 g) and a screwdriver with an irregular surface and unbalanced mass distribution (diameter: 14 mm, 255 g) shown in Figure 8d. All of these objects were grasped using the pinching mode. Fragile objects such as a potato chip (diameter: 50 mm, thickness: 0.8 mm, 2 g) and tofu (diameter: 43 mm, 30 g) were grasped, as shown in Figure 8e. Additionally, a soft doll with an irregular shape (diameter: 58 mm, 47 g) and a big bottle of water (diameter: 58 mm, 542 g, about 10 times the gripper's own weight) were grasped, as shown in Figure 8f. These objects all use the enveloping mode. A hollow box (diameter: 58 mm, 14 g) was picked up using the expanding mode, as shown in Figure 8g. This mode can be combined with different actuation modes and driving steps to achieve variable grasping heights of the gripper. Additionally, a soft and fragile cherry was successfully grasped and rotated using the kneading mode, as shown in Figure 8h. The results validate the soft gripper's adaptivity to objects with various physical characteristics, through appropriate grasping modes. The gripper's diverse grasping modes are achieved through the combination of the SCOPs’ actuation modes, validating the practicality of the actuator's multidimensional motions in enhancing the manipulation capability of the soft robotic gripper. The grasping experiments are recorded in Movie S3 (Supporting Information) in the Supplementary Information.

### 4.3 Single‐Actuator Crawling Robot

**Figure 9:**
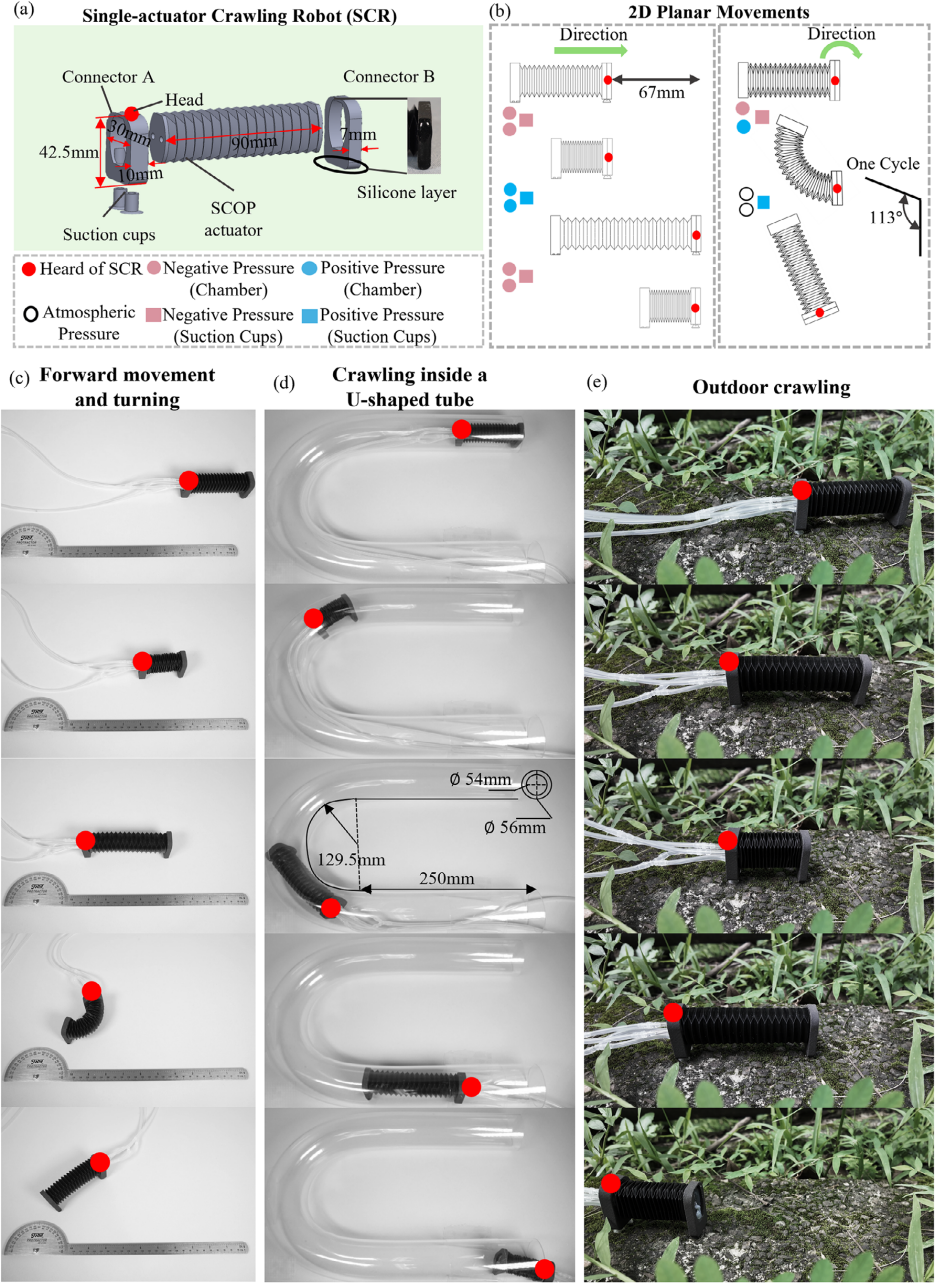
Demonstrations of the SCR. a) The robot was constructed using a single SCOP actuator. b) The 2d planar movements of the SCR. c) The forward movement and turning of the SCR. d) The SCR moves within an acrylic U‐shaped tube with an inner diameter of 54 mm. e) The SCR can move in complex outdoor environments.

To demonstrate the SCOP actuator's stand‐alone capability in soft robots, a single‐actuator soft crawling robot (SCR) was developed (**Figure** **9**a). Enabled by active elongation, contraction, bidirectional bending, and passive compliance, the SCR achieves two‐dimensional planar locomotion. It moves by generating asymmetric friction through an active and passive suction mechanism on Connector A and a friction‐enhancing layer on Connector B. This configuration allows both linear and turning motions under simple control and provides adaptability across various environments.

The SCR is pneumatically controlled by three pneumatic input channels. Two channels independently regulate the dual chambers of the SCOP actuator, and the third controls the suction cups. Figure 9b shows the actuation modes, where the labels of circles indicate the actuator chambers and squares correspond to the suction cup control. Benefiting from the large‐range motions of the SCOP actuator, the SCR achieves long stride forward locomotion by coordinating the I–I and D–D actuation modes in conjunction with the alternating anchoring of the suction cups. The detailed one‐cycle of the forward stride is illustrated in Figure 9b. Each cycle of motion results in a forward distance of ≈67 mm, which corresponds to ≈74% of the SCR's initial body length. Using the I–D actuation mode of the SCOP actuator, the SCR is capable of executing turning movements, achieving a turning angle of 113° in a single stride. Compared with the existing soft crawling robots as listed in **Table** **2**, SCR demonstrates superior stride length in forward locomotion and larger turning angles, highlighting the advantage of the SCOP in obtaining large‐range motions.

**Table 2:** Comparison with existing crawling robots.

	Ref. [18]	Ref. [57]	Ref. [61]	Ref. [62]	SCR
Initial length (mm)	68	105	43	198	90
Forward Step Length (mm)	33.6	25	17.9	22.5	67
Turning Step Angle (degree)	92	45	20	15	113

To evaluate the versatility and operational capability of the SCR, we conducted performance tests in various environments. As shown in Figure 9c, the SCR achieved 2D planar locomotion on a smooth surface. Demonstration process performed a forward displacement of 164 mm and a turning angle of 290° was achieved. This locomotion performance verifies the advantage of the actuator's large‐range motions and coordinated actuation modes.

The U‐shaped pipe crawling test (Figure 9d) highlights the structural compliance of the SCR in constrained geometries. By utilizing its inherent flexibility through environment‐adaptive deformation and active actuation, the robot successfully traversed a U‐shaped pipe with an inner diameter of 54 mm while maintaining consistent motion efficiency. This test validates the passive adaptability provided by the intrinsic compliance of the SCOP actuator when interacting with complex environments, and verifies the structural stability during continuous interaction with the confined tubular structure. An outdoor crawling test was conducted on a vegetation substrate, as shown in Figure 9e. The results demonstrate the SCR's strong environmental adaptability. The robot successfully traversed a 620 mm uneven terrain composed of mud and vegetation obstacles, confirming its effective mobility in complex outdoor environments. The entire process was recorded in the supplementary Movie S4 (Supporting Information).

## 5 Conclusion and Future Work

In this work, we propose a straight–curved crease dual‐chamber prismatic design for SPAs that enables large‐range motions with enhanced structural stability. The integration of straight and curved origami creases induces specific anisotropic lateral stiffness, contributing to structural reinforcement while enabling flexible 2‐DOF motions. A foldable partition divides the inner chamber and simultaneously enhances structural stability, allowing contraction, extension, and bidirectional bending under both positive and negative pressure inputs. The SCOP design enables one‐step fabrication of the dual‐chamber prismatic structure via a molding and casting process using a single material. A wall thickness–airtightness failure risk function is derived to guide the manufacturing process and achieve airtightness. By inflating and deflating the inner chambers under different actuation modes (I–A, D–A, I–D, I–I, and D–D), the SCOP actuator generates antagonistic or synergistic interactions, enabling multidimensional motions that have been demonstrated in soft robotic applications.

To characterize the motions and deformation behaviors of the SCOP actuator, mechanical and kinematic models were established and experimentally validated. The effectiveness of the structural reinforcement is further verified through FEM. As a result, the proposed SCOP actuator, with an initial length of 90 mm, achieves an axial displacement of up to 106.7% through 46 mm of elongation and 50 mm of contraction, along with a maximum bending angle of 160°. This demonstrates a significantly larger motion range compared to state‐of‐the‐art soft actuators, as summarized in Table 1. The structural stability is increased by up to 209% compared to curve crease bellows actuator and 51% compared to straight crease origami actuator.

Based on the versatile motion capabilities of the SCOP actuator, three representative robotic applications were developed. A versatile wearable assistive device for hand joints was designed, which can assist different joints simply by changing its wearing position. By utilizing various actuation modes of the SCOP actuator, the device is capable of covering the full range of motion required by the thumb and wrist joints. A two‐finger soft gripper was developed using the same actuator principle, enabling diverse grasping motions through different actuation modes. The gripper's performance was experimentally validated by successfully grasping objects with a wide range of surface characteristics and dimensions, from as small as 0.6 to 85 mm in size and from 0.1 g to 542 g in weight. These results demonstrate that multidimensional motion significantly enhances the operational versatility and control capability of soft robotic grippers. Equipped with a single SCOP actuator, the SCR robot leverages its large‐range motion capability and the inherent compliance of soft actuators to achieve superior performance compared to similar crawling robots (as summarized in Table 2). It demonstrates 2D planar omnidirectional locomotion in confined and unstructured environments, achieving a step length of ≈67 mm, which corresponds to ≈74% of its initial body length, and a turning angle of up to 113° in a single actuation step. The robot also successfully traverses a U‐shaped pipe and completes outdoor crawling tests on a vegetation substrate, demonstrating strong adaptability to complex environments.

These experimental results validate the effectiveness of the proposed SCOP design in constructing soft actuators that combine large‐range, multidimensional motions with structural stability. The SCOP actuator shows strong potential for application across diverse soft robotic scenarios. Serving as a modular unit with integrated functionalities, it paves the way for the development of more flexible and intelligent soft robotic systems. One current limitation lies in the trade‐off between motion range and load‐bearing capacity; achieving large‐range deformation inevitably compromises the actuator's ability to handle high payloads, which may restrict its use in tasks requiring strong force output. Future research will focus on integrating multiple actuator modules and incorporating sensing capabilities to further enhance the system's intelligence and environmental adaptability.

## Conflict of Interest

The authors declare no conflict of interest.

## Supporting information



Supporting Information

Supplemental Movie 1

Supplemental Movie 2

Supplemental Movie 3

Supplemental Movie 4

## Data Availability

The data that support the findings of this study are available from the corresponding author upon reasonable request.

## References

[1] D. Rus , M. T. Tolley , Nat. Rev. Mater. 2018, 3, 101.

[2] Z. Ye , L. Zheng , W. Chen , B. Wang , L. Zhang , Adv. Mater. Technol. 2024, 2301862.

[3] R. Pfeifer , M. Lungarella , F. Iida , Science 2007, 318, 1088. 18006736 10.1126/science.1145803

[4] O. Yasa , Y. Toshimitsu , M. Y. Michelis , L. S. Jones , M. Filippi , T. Buchner , R. K. Katzschmann , Annu. Rev. Control Rob. Auton. Syst. 2023, 6, 1.

[5] B. Gorissen , D. Reynaerts , S. Konishi , K. Yoshida , J.‐W. Kim , M. De Volder , Adv. Mater. 2017, 29, 1604977. 10.1002/adma.201604977 28949425

[6] M. Li , A. Pal , A. Aghakhani , A. Pena‐Francesch , M. Sitti , Nat. Rev. Mater. 2022, 7, 235. . 35474944 10.1038/s41578-021-00389-7 PMC7612659

[7] N. El‐Atab , R. B. Mishra , F. Al‐Modaf , L. Joharji , A. A. Alsharif , H. Alamoudi , M. Diaz , N. Qaiser , M. M. Hussain , Adv. Intell. Syst. 2020, 2, 2000128. .

[8] H. Kawasaki , S. Ito , Y. Ishigure , Y. Nishimoto , T. Aoki , T. Mouri , H. Sakaeda , M. Abe , in 2007 IEEE 10th International Conference on Rehabilitation Robotics , IEEE, Noordwijk, Netherlands 2007, pp. 234–240.

[9] J. Shintake , V. Cacucciolo , D. Floreano , H. Shea , Adv. Mater. 2018, 30, 1707035. 10.1002/adma.201707035 29736928

[10] C. Majidi , Soft Rob. 2014, 1, 5.

[11] S. Liu , F. Chen , D. Duanmu , Y. Wang , J. Liu , W. Yang , Y. Zhu , Y. Wu , J. Yi , J. S. Dai , Z. Wang , Chem. Eng. J. 2024, 489, 151462.

[12] J. Auysakul , A. Booranawong , N. Vittayaphadung , P. Smithmaitrie , Actuators 2023, 12, 300.

[13] M. Feng , D. Yang , L. Ren , G. Wei , G. Gu , Sci. Adv. 2023, 9, adi7133. 10.1126/sciadv.adi7133 PMC10511197 37729399

[14] Z. Wang , P. Polygerinos , J. T. B. Overvelde , K. C. Galloway , K. Bertoldi , C. J. Walsh , IEEE/ASME Trans. Mechatron. 2017, 22, 717.

[15] D. Li , D. Fan , R. Zhu , Q. Lei , Y. Liao , X. Yang , Y. Pan , Z. Wang , Y. Wu , S. Liu , H. Wang , Soft Rob. 2023, 10, 395. 10.1089/soro.2021.0185 36318818

[16] D. R. Yao , I. Kim , S. Yin , W. Gao , Adv. Mater. 2024, 36, 2308829. 10.1002/adma.202308829 38305065

[17] A. Ainla , M. S. Verma , D. Yang , G. M. Whitesides , Soft Rob. 2017, 4, 297. 10.1089/soro.2017.0017 29182081

[18] Z. Fang , S. Tang , Y. Su , X. Liu , S. Liu , J. Yi , Z. Wang , J. S. Dai , Adv. Sci. 2025, 12, 2409060. 10.1002/advs.202409060 PMC11744560 39587985

[19] M. A. Bell , B. Gorissen , K. Bertoldi , J. C. Weaver , R. J. Wood , Adv. Intell. Syst. 2022, 4, 2100094.

[20] Y. X. Mak , A. Dijkshoorn , M. Abayazid , Adv. Intell. Syst. 2024, 6, 2300666.

[21] K. F. Hussain , W. Cantwell , K. A. Khan , Int. J. Mech. Sci. 2024, 284, 109724.

[22] K. Song , H. Li , Y. Li , J. Ma , X. Zhou , Front. Phys. 2024, 12.

[23] B. Chen , Z. Shao , Z. Xie , J. Liu , F. Pan , L. He , L. Zhang , Y. Zhang , X. Ling , F. Peng , W. Yun , L. Wen , Adv. Intell. Syst. 2021, 3, 2000251.

[24] G. W. Hunt , I. Ario , Int. J. Non‐Linear Mech. 2005, 40, 833.

[25] W. Kim , J. Eom , K.‐J. Cho , Adv. Intell. Syst. 2022, 4, 2100176.

[26] E. D. Demaine , J. O'Rourke , Geometric Folding Algorithms: Linkages, Origami, Polyhedra, Cambridge University Press, Cambridge, UK 2007.

[27] K. Higashi , K. Koyama , R. Ozawa , K. Nagata , T. Kiyokawa , W. Wan , K. Harada , IEEE Access 2023, 11, 129258.

[28] D. Duanmu , X. Wang , X. Li , Z. Wang , Y. Hu , Actuators 2022, 11, 346.

[29] C.‐Y. Yeh , S.‐C. Chou , H.‐W. Huang , H.‐C. Yu , J.‐Y. Juang , Extreme Mech. Lett. 2019, 26, 61.

[30] H. Ma , J. Zhou , Acta Mech. Solida Sin. 2023, 36, 1.

[31] D. Liu , S. Liu , W. Yang , J. Yi , H. Wang , W. Zhang , J. S. Dai , Z. Wang , Adv. Intell. Syst. 2024, 6, 2300767.

[32] S. Liu , J. Liu , K. Zou , X. Wang , Z. Fang , J. Yi , Z. Wang , J. Mech. Rob. 2022, 14, 060912.

[33] M. Park , W. Kim , S.‐Y. Yu , J. Cho , W. Kang , J. Byun , U. Jeong , K.‐J. Cho , IEEE Rob. Autom. Lett. 2023, 8, 864.

[34] Y. Su , Z. Fang , W. Zhu , X. Sun , Y. Zhu , H. Wang , K. Tang , H. Huang , S. Liu , Z. Wang , IEEE Rob. Autom. Lett. 2020, 5, 3003.

[35] W. Chen , C. Xiong , C. Liu , P. Li , Y. Chen , Soft Rob. 2019, 6, 495. 10.1089/soro.2018.0061 PMC6690731 30907704

[36] Y. Wu , X. Liu , S. Tang , S. Liu , J. Yi , Z. Wang , J. S. Dai , in *2024 6th International Conference on Reconfigurable Mechanisms and Robots* (ReMAR), IEEE, Chicago, IL, USA 2024, pp. 601–607.

[37] Q. Tan , Y. Chen , J. Liu , K. Zou , J. Yi , S. Liu , Z. Wang , Front. Robot. AI 2021, 8. 10.3389/frobt.2021.688697 PMC8427137 34513936

[38] L.‐J. Gai , J. Huang , X. Zong , IEEE/ASME Trans. Mechatron. 2023, 28, 2897.

[39] W. Zhu , C. Lu , Q. Zheng , Z. Fang , H. Che , K. Tang , M. Zhu , S. Liu , Z. Wang , IEEE/ASME Trans. Mechatron. 2023, 28, 104.

[40] S. Chai , Z. Hu , Y. Chen , Z. You , J. Ma , J. Mech. Phys. Solids 2024, 193, 105877.

[41] A. Miriyev , K. Stack , H. Lipson , Nat. Commun. 2017, 8, 596. 28928384 10.1038/s41467-017-00685-3 PMC5605691

[42] C. Duriez , in 2013 IEEE International Conference on Robotics and Automation , IEEE, Karlsruhe, Germany 2013, pp. 3982–3987.

[43] J. Liu , X. Wang , S. Liu , J. Yi , X. Wang , Z. Wang , IEEE Robot. Autom. Lett. 2022, 7, 658.

[44] N. S. Usevitch , Z. M. Hammond , M. Schwager , A. M. Okamura , E. W. Hawkes , S. Follmer , Sci. Robot. 2020, 5, aaz0492. 10.1126/scirobotics.aaz0492 33022597

[45] S. Liu , Y. Zhu , Z. Zhang , Z. Fang , J. Tan , J. Peng , C. Song , H. H. Asada , Z. Wang , IEEE/ASME Trans. Mechatron. 2021, 26, 2747.

[46] S. Wang , P. Zhang , L. He , P. Maiolino , Soft Rob. 2024, 11, 617. 10.1089/soro.2023.0049 39178399

[47] S. Lin , R. Sun , T. Jiang , D. Zhang , Y. Sun , J. Phys.: Conf. Ser. 2025, 2954, 012042.

[48] W. Zhou , Y. Li , Soft Rob. 2020, 7, 168. 10.1089/soro.2018.0087 32255420

[49] Y. Zhu , K. Chu , X. Chen , X. Wang , H. Su , Sens. Actuators, A 2022, 338, 113492.

[50] H. Li , J. Yao , P. Zhou , X. Chen , Y. Xu , Y. Zhao , Sens. Actuators, A 2020, 306, 111957.

[51] A. A. Amiri Moghadam , S. Alaie , S. Deb Nath , M. Aghasizade Shaarbaf , J. K. Min , S. Dunham , B. Mosadegh , Soft Rob. 2018, 5, 443. 10.1089/soro.2017.0069 PMC6094352 29924697

[52] R. Natividad , M. Del Rosario , P. C. Y. Chen , C.‐H. Yeow , Soft Rob. 2018, 5, 304. 10.1089/soro.2017.0064 29883297

[53] M. A. Bell , K. P. Becker , R. J. Wood , Adv. Mater. Technol. 2022, 7, 2100605.

[54] S. P. Sutera , R. Skalak , Annu. Rev. Fluid Mech. 1993, 25, 1.

[55] J. J. He , N. Li , N. Tang , X. Y. Wang , C. Zhang , L. Liu , Intermetallics 2012, 21, 50.

[56] V. Ferrandy , Indrawanto, F. F. , A. Sugiharto , E. Franco , A. Garriga‐Casanovas , A. I. Mahyuddin , F. Rodriguez Y Baena , S. Mihradi , V. Virdyawan , Robotica 2023, 41, 3608.

[57] M. Su , R. Xie , Y. Qiu , Y. Guan , J. Bionic Eng. 2023, 20, 69.

[58] J. Yoon , J. Yang , D. Yun , IEEE Rob. Autom. Lett. 2024, 9, 4567.

[59] J. C. Becker , N. V. Thakor , IEEE Trans. Biomed. Eng. 1988, 35, 110. 3350537 10.1109/10.1348

[60] A. Chaparro , M. Rogers , J. Fernandez , M. Bohan , C. Sang Dae , L. Stumpfhauser , Disability Rehabil. 2000, 22, 633. 10.1080/09638280050138313 11052213

[61] Q. Xu , K. Zhang , C. Ying , H. Xie , J. Chen , S. E. , Biomimetics 2024, 9, 541. 39329563 10.3390/biomimetics9090541 PMC11430112

[62] Y. Zhang , Y. Li , D. Sui , L. Luan , T. Zheng , Z. Zhang , S. Zhao , F. Zhang , D. Li , Y. Zhu , Soft Rob. 2025. 10.1089/soro.2025.0002 40353772

