# Supplementary material for: Dual‐Chamber One‐Step Molding Actuator with Straight‐Curved Crease Prismatic Design for Large‐Range Motions and Structural Stability
Source: Adv Sci (Weinh). 2025 Nov 20;13(2):e14441. doi: 10.1002/advs.202514441 (PMC12786339; doi:10.1002/advs.202514441)
Supplement: Supplementary file 1 — Supporting Information [file ADVS-13-e14441-s004.pdf]

Supplementary Information

Dual-chamber One-step Molding Actuator with Straight-curved Crease Prismatic Design for Large-range Motions and Structural Stability

Qinlin Tan, Yanlin Chen, Meng Zhou, Shuk Fan Tong, Sicong Liu, and Raymond Kai-Yu Tong**

Q. Tan, M. Zhou, S. Tong, R. K. Tong

Department of Biomedical Engineering

The Chinese University of Hong Kong

Hong Kong SAR, China

E-mail: kytong@cuhk.edu.hk

Y. Chen

Department of Mechanical and Automation Engineering

The Chinese University of Hong Kong

Hong Kong SAR, China

S. Liu

Sino-German College of Intelligent Manufacturing

Shenzhen Technology University

Pingshan 518118, China

E-mail: liusicong@sztu.edu.cn

Keywords: (soft actuator, origami, soft robot, one-step molding)

This document contains:

Note S1. Derivation of effective cross-sectional area

Note S2. Modeling of linear motions

Note S3. Modeling of bending motions

Note S4. Kinematic model and SCOP's theoretical linear range of motions

Note S5. SCOP's theoretical linear range of motions

Note S6. Material Characterization and FEM Setup

Note S7. Experimental Setups

Note Figure S1. Material properties and FEM fitting results of the SCOP actuator

Note Table S1. Geometry parameters of the CCB actuator and the SCO actuator.

Note Table S2. Geometry parameters of the SCOP actuator prototype

Movie S1. The one-step molding process of the SCOP actuator

Movie S2. Versatile soft wearable assistive device

Movie S3. Demonstrations on multimodal two-finger soft gripper

Movie S4. Single-actuator crawling robot

Note S1. Derivation of effective cross-sectional area

The effective cross-sectional area S_a is a key parameter in deriving the actuator's output force, displacement, and bending angle. Since the prismatic cross-section features a convex circular shape that is difficult to model analytically, an elliptical approximation is employed to assist with the derivative process. As shown in Figures 2a-2c, the cross-sectional characteristics of the SCOP actuator consist of two parts A-A and B-B as shown in Figure 2c. Based on geometric analysis, the o-xyz coordinate system is established. S_a is the equivalent cross-section area of the actuator, which can be assumed to be $\frac{(S_1+S_2)}{2}$, as shown in Figure 2c, and it is assumed that the equivalent cross-sectional area remains unchanged during motions[1]. According to the geometric relationship between A-A and B-B, S_a can be described as

$$S_a = \frac{\pi ab}{8} + \frac{\pi a'b'}{8} - \frac{\varepsilon l_y l_c}{2}\sqrt{\left(\frac{b}{2}\right)^2 \cdot \left[1 - \frac{\left(\frac{a}{2} - l_c\right)^2}{\left(\frac{a}{2}\right)^2}\right]} - \frac{\xi l_y' l_c'}{2} \tag{1}$$

where a and b denote the lengths of the major and minor axes of the ellipse E_1, respectively, while a' and b' represent the lengths of the major and minor axes of the ellipse E_2, respectively. The parameters ε and ξ denote the area ratio of triangle DEF within the closed region CEDF, and the area ratio coefficient of triangle GMJ within the enclosed region GMJC′, respectively. l_c and l_c' are the lengths of the line segments CD and MC′, respectively, and l_y and l_y' are the lengths of the line segments EC and GC′, respectively, as shown in Figure 2c. These parameters govern the cross-sectional design of the actuator, thereby determining the widths of the CCB facets and the SCO facet of the structure, and define the prismatic shape.

Note S2. Modeling of linear motions

The model is derived based on the fundamental equation of force under fluid pressure, i.e., $F = PS$, where P is the internal pressure, and S is the effective force-bearing area. Based on S_a, the statics models focus on the axial linear motion of the actuator can be obtained, which establish the relationship among input pressure, output force, and displacement. During the axial motion of the actuator, the output force F consists of two components. The first is generated by internal pressure P. The second is the elastic restoring force resulting from the intrinsic properties of the soft materials and the actuator's foldable structural design,[2] which is characterized by a nonlinear stiffness that varies with the axial displacement ΔH_z. Therefore, the output force can be expressed by

$$F = F_d(P) + F_a(\Delta H_z) = PS_a + k(\Delta H_z)\Delta H_z \quad (2)$$

where S_a is the effective cross-sectional area (Note S1. Supporting Information), F is the output force of the actuator during axial motion, $F_d(P)$ is output force generated by the input air pressure P, and $k(\Delta H_z)$ is the axial stiffness of the actuator during displacement ΔH_z, which can be obtained through experiments and numerical methods using nonlinear fitting. Then, by substituting Eqn. (1) into (2), the axial output force F can be developed by

$$F = \left(\frac{\pi ab}{8} + \frac{\pi a'b'}{8} - \frac{\varepsilon l_y l_c}{2} \sqrt{\left(\frac{b}{2}\right)^2 \cdot \left[1 - \frac{\left(\frac{a}{2} - l_c\right)^2}{\left(\frac{a}{2}\right)^2} \right]} - \frac{\xi l_y' l_c'}{2} \right) P + k(\Delta H_z)\Delta H_z \quad (3)$$

Eqn. (3) establishes the relationship between the axial output force and the input pneumatic pressure when the actuator interacts with the external environment. Consequently, this relationship can be effectively used to guide control inputs and estimate the axial output force when deploying the actuator in practical applications.

When no external force other than input pneumatic pressure is considered, $F_a(H_z)$=$F_d(P)$. Under this condition, the relationship between the actuator's linear displacement and the variation in internal pressure can be derived as follows:

$$\Delta H_z = \frac{S_a}{k(\Delta H_z)} \cdot P \quad (4)$$

Eqn. (4) describes the axial motion of the SCOP actuator under pneumatic actuation.

Note S3. Modeling of bending motions

To describe the bending behaviors of the SCOP soft actuator, the relationship between the chamber pressures P_1 and P_2 and the resulting bending angle ω is analyzed. Specifically, to establish the correlation among the bending angle, lateral output force, and internal pressures during bending motions, a geometric analysis is conducted. Based on this analysis, the following results are derived as

$$\omega = 2\sin^{-1}\left(\frac{\left(S_a\left(l_p + l_q\right)\right)}{8H_0 l_n H_{pw}} \cdot \left(\frac{P_1}{k(\Delta l_1)} - \frac{P_2}{k(\Delta l_2)} \right) \right) \quad (5)$$

where l_p and l_q are the outer and inner widths of the D-D cross-section in the folded partition, respectively, as shown in Figure 2e. H_{pw} is the central arc length of the folded partition. $k\ (\Delta l_1)$ and $k\ (\Delta l_2)$ are the stiffnesses of the two cavities at axial displacements of Δl_1 and Δl_2, respectively, which can be experimentally determined. The parameter l_n denotes the central

distance from each of the two chambers to the actuator's central axis, serving as a geometric reference for determining the width of each chamber, as shown in Figure 2f.

In applications requiring large-range motions, such as finger-wearable devices and soft grippers, interaction with the user or environment often occurs through the lateral surface at the actuator's tip. Therefore, deriving the model of the lateral tip force is essential for understanding the actuator's interactive performance. The tip force of the SCOP actuator consists of two components: the lateral force induced by the applied pneumatic pressure, and the restoring lateral force generated during bending. The relevant geometrical parameters are illustrated in Figures 2e and 2f. When the two chambers of the SCOP actuator, AC-1 and AC-2, are subjected to respective pneumatic pressures P_1 and P_2, forces F_1 and F_2 on the end surfaces are produced. The difference between these forces generates a torque M about the actuator's neutral axis. Based on the geometric and static analysis, the resulting lateral tip force F_S can be expressed as:

$$F_S = F_b(P_1, P_2) + F_b(\omega) = \frac{(P_1 - P_2) \cdot S_a \cdot l_n \cdot \cos\left(\frac{\omega}{2}\right)}{4(H_0 + \Delta l_2 + l_n \cdot \sin(\omega/2))} + \frac{6k(\Delta H_z) I \sin\omega}{S_a H_{pw}} \quad (6)$$

where $F_b(P_1, P_2)$ is the lateral force generated under P_1 and P_2 when the actuator bends to an angle ω, and $F_b(\omega)$ is the restoring lateral force due to bending stiffness at angle ω, which can be derived based on the experimentally fitted axial stiffness $k(\Delta H_z)$. The second moment of inertia I, corresponding to the SCOP actuator bending along the CCB facets, can be directly calculated from the geometry of the design.

Note S4. Kinematic model and SCOP's theoretical linear range of motions

The kinematic model is established to estimate the spatial orientation and position of the center point of the end panel, the actuator space $[H_{pw}, \omega]$is mapped to the task space, obtaining the transformation matrix $\boldsymbol{T}$ with rotation matrix $\boldsymbol{R_x}$ and position matrix $\boldsymbol{P} = [x, y, z]^{\mathrm{T}}$ as follows:

$$\boldsymbol{T} = \begin{bmatrix} \boldsymbol{R}_x & \boldsymbol{P} \\ \boldsymbol{O} & \boldsymbol{I} \end{bmatrix} = \begin{bmatrix} \cos\omega & 0 & \sin\omega & H_{pw}/\omega(1-\cos\omega) \\ 0 & 1 & 0 & 0 \\ -\sin\omega & 0 & \cos\omega & H_{pw}/\omega\sin\omega \\ 0 & 0 & 0 & 1 \end{bmatrix} \quad (7)$$

Note S5. SCOP's theoretical linear range of motions

The design of the folded partition influents the actuator's axial extension and contraction range. H_{pw} has a limited range throughout the folding and unfolding process, which does not involve material stretching or compression. According to the geometric relationship

$$2N_a l_{tf} \leq H_{pw} \leq \frac{2N_a}{W_i}\sqrt{\left(\frac{hW_i}{2}\right)^2 + {l_m}^2} \tag{8}$$

where l_{tf} is the thickness of the folded partition, W_i is the number of folded layers that each folded partition spans across i layer of the outer wall, and h is the height of each outer wall layer, as shown in Figure 2d.

Note S6. Material Characterization and FEM Setup

Based on a literature review of simulation methods for soft actuators [3,4] and recent advances in the field [5–7], the finite element method (FEM) analysis of the SCOP actuator was conducted using a fully explicit model in Abaqus/Explicit. To obtain accurate material properties and ensure consistency between simulation and experimental data, tensile tests were performed using Dogbone specimen made of Hei-Cast 8400 (Shore hardness 60A) on a computer-controlled testing machine (Figure S2a). The nominal stress–strain curve obtained prior to failure is shown in Figure S2b, with a maximum strain reaching 320%, indicating hyperelastic behavior. Accordingly, the Yeoh model was selected for the FEM material definition in Abaqus, in line with the modeling approaches reported in [8]. Its strain energy function can be expressed as:

$$W = \sum_{i=1}^{3} C_{i0}(I_1 - 3)^3$$

where W denotes the strain energy, I_1 denotes the first deviatoric strain invariant, and C_{i0} is the material constant. The results are imported into ABAQUS for material coefficient fitting, so that the material constants of the third-order Yeoh model can be obtained as follows: $C_{10} = 1.402$ MPa , $C_{20} = -1.322 \times 10^{-2}$ MPa , $C_{30} = 9.514 \times 10^{-4}$ Mpa. The fitting results obtained using the Yeoh model in Abaqus are shown in Figure S2b, with the R-squared Coefficient of Determination (R^2) as high as 0.997. This further confirms the validity and effectiveness of defining the SCOP actuator material as a hyperelastic body and applying the Yeoh model for FEM simulation.

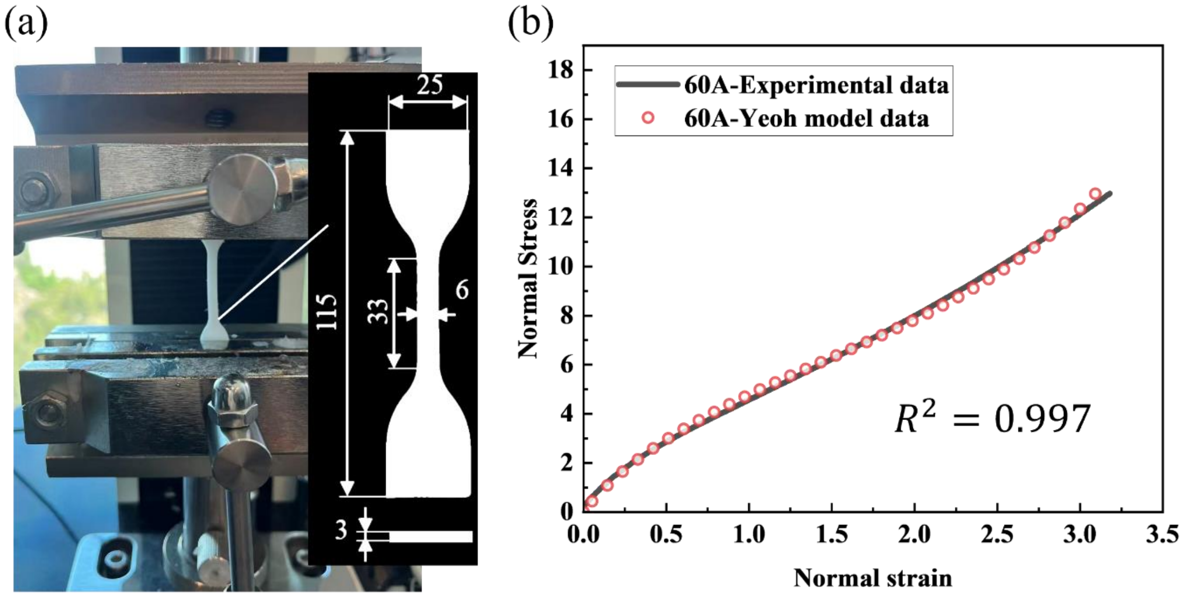

Figure S1. Material properties of the SCOP actuator. a) Uniaxial tensile test of Shore hardness 60A Hei-Cast 8400 Dogbone specimen. b) The simulation results on the tensile test are compared with the experiment that validated the material properties.

Note S7. Experimental Setups

Figure 5a shows the linear motion platform. During the experiment, soft actuator moves along the slide rail (RXP45-1000, Ruixin), which ensures uniform axial motion. A laser displacement sensor (HG-C1400, Panasonic) and a force sensor (DYMH-103, DAYSENSOR) are used to record displacement and output force, respectively. Figure 5b presents the bidirectional bending experiment platform. An inertial measurement unit (IMU, JY61, WitMotion) attached to the actuator captures the bending angle. Both setups integrate a microcontroller (STM32F103C8T6) and two pressure sensors (XGZP6847A, 400 kPa, CFSensor) for real-time data acquisition through an ADC module and serial communication. Data is processed using MATLAB (R2023b, MathWorks) and Origin 2024. Supporting components were fabricated using 3D printing with material of polylactic acid (PLA).

A pneumatic actuation system is developed to provide the air source and is connected to the experimental device, as shown in Figure 5c. The actuation system consists of two pumps (HLVP8-WB12,12W, Kamoer Inc.), a set of solenoid valves (Solenoid T103U-FG 12V 0.9W, OST Inc.), a microcontroller (Arduino Mega 2560) embedded with the control algorithm, and pressure sensors (XGZP6847A, -100kPa-300kPa, CFSensor Inc.). It is powered by an external battery or a 12V DC power adapter and equipped with manual switches to obtain individual control of the actuators.

Note Table S1. Geometry parameters of the CCB actuator and the SCO actuator.

Design	H_0 (mm)	h (mm)	a (mm)	b (mm)	l_c (mm)	l_t (mm)	α	β	S_a (mm^2)
CCB actuator	90	5	35	20	5	0.8	60°	60°	670.35
SCO actuator	90	5	35	20	5	0.8	60°	60°	619.42

Note Table S2. Geometry parameters of the SCOP actuator prototype.

Geometry parameter	Value	Geometry parameter	Value
Height of soft actuators H	94 mm	Major axis of ellipse a	35 mm
Height of the folded section H_0	90 mm	Minor axis of ellipse b	20 mm
Thickness of soft actuators l_t	0.8 mm	Dihedral angle of origami element α	60°
Height of each layer h	5 mm	Number of outer wall fold layers N_a	16
Distance between the origami face and the vertex of the ellipse l_c	5 mm	Each layer of the folded partition spans the external folded layers W_i	2
Dihedral angle of bellows element β	60°	The width of each chamber l_n	30 mm
Effective cross-sectional area S_a	630.53 mm^2	The thick of folded partition l_{tf}	0.8 mm
The width of folded partition l_m	6.12 mm		

References

[1] W. Zhou, Y. Li, *Soft Robotics* **2020**, *7*, 168.

[2] Y. Su, Z. Fang, W. Zhu, X. Sun, Y. Zhu, H. Wang, K. Tang, H. Huang, S. Liu, Z. Wang, *IEEE Robotics and Automation Letters* **2020**, *5*, 3003.

[3] M. S. Xavier, A. J. Fleming, Y. K. Yong, *Advanced Intelligent Systems* **2021**, *3*, 2000187.

[4] A. Pagoli, F. Chapelle, J.-A. Corrales-Ramon, Y. Mezouar, Y. Lapusta, *Smart Mater. Struct.* **2021**, *31*, 013001.

[5] A. R. Elchrif, M. I. Awad, S. A. Maged, A. Ramzy, *Sci Rep* **2024**, *14*, 24169.

[6] W. Xiao, C. Xie, Q. Liu, K. Wang, W. Zhu, L. Zeng, *Smart Mater. Struct.* **2025**, *34*, 055014.

[7] X. Yang, T. Jin, S. Tian, J. Wang, S. Yi, Y. Wang, L. Li, Y. Lin, *Advanced Science* **2025**, *12*, 2501477.

[8] O. H. Yeoh, *Rubber Chemistry and Technology* **1993**, *66*, 754.
